# DNA-Programmable Protein Degradation: Dynamic Control of Proteolysis-Targeting Chimera Activity via DNA Hybridization and Strand Displacement

**DOI:** 10.1021/jacsau.5c00422

**Published:** 2025-07-31

**Authors:** Disha Kashyap, Shozeb Haider, Thomas A. Milne, Michael J. Booth

**Affiliations:** † Department of Chemistry, 6396 University of Oxford, Mansfield Road, Oxford OX1 3TA, U.K.; ‡ MRC Molecular Haematology Unit, MRC Weatherall Institute of Molecular Medicine, Radcliffe Department of Medicine, University of Oxford, Oxford OX3 9DS, U.K.; § UCL School of Pharmacy, 4919 University College London, London WC1N 1AX, U.K.; ∥ University of Tabuk (PFSCBR), Tabuk 47512, Saudi Arabia; ⊥ UCL Centre for Advanced Research Computing, University College London, London WC1H 9RL, U.K.; # Department of Chemistry, University College London, 20 Gordon Street, London WC1H 0AJ, U.K.

**Keywords:** PROTACs, oligonucleotide-linked PROTACs, DNA nanotechnology, toehold-mediated strand displacement, BRD4 degradation, VHL E3 ligase, JQ1, VH032

## Abstract

Targeted protein degradation is a powerful therapeutic approach: expanding the druggable proteome, providing enhanced selectivity, and having the ability to overcome conventional resistance mechanisms. A major class of such molecules is proteolysis-targeting chimeras (PROTACs). PROTACs are catalytic heterobifunctional small molecules that simultaneously bind a protein of interest (POI) and an E3 ligase. And thus, PROTACs induce a proximity-dependent ubiquitination of the POI and its subsequent degradation by the ubiquitin–proteasome system. While PROTACs have successfully transitioned from academia to industry, increasing awareness of off-target effects and related toxicities highlights the urgent need for precise control mechanisms over activity. Achieving this level of control, however, remains challenging, with traditional chemistries. DNA nanotechnology, with its unparalleled programmability and structural versatility, presents a powerful tool for achieving such control. Here, we report the design and characterization of oligonucleotide-linked PROTACs (OligoPROTACs). These constructs comprise PROTAC warheads covalently linked to separate complementary DNA strands, brought together in space via DNA hybridization. OligoPROTACs are able to degrade the POI in a distance-dependent manner. Furthermore, we demonstrate the first instance of a dynamic off-switch mechanism for PROTAC activity, enabled by toehold-mediated strand displacement using a third DNA strand. This work highlights the potential of DNA nanotechnology combined with the clinical emergence of nucleic acid therapeutics to enhance the safety and functionality of PROTAC systems, paving the way for more refined and translatable therapeutic strategies.

## Graphical abstract

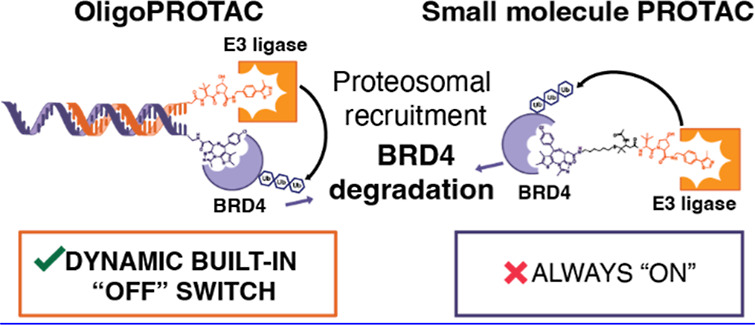

## Introduction

Targeted protein degradation has emerged as a transformative approach in drug discovery.^1^ Protein degradation offers significant advantages over protein inhibition: long-lasting therapeutic effects upon removal of the target protein, lower drug concentrations for achieving therapeutic activity, and a wider array of protein targets. Unlike inhibition, which is limited to the presence of a specific functional domain or active site in a protein, degradation has no such constraints: expanding the druggable proteome. One of the most promising class of molecules for targeted protein degradation is proteolysis-targeting chimeras (PROTACs).^2,3^ These catalytic, heterobifunctional small molecules simultaneously bind a protein of interest (POI) and an E3 ligase.^4^ This binding induces a proximity-dependent ubiquitination of the POI by the E3 ligase, marking it for degradation by the ubiquitin–proteasome system.^5^ Thereby, hijacking a natural cellular process responsible for maintaining protein homeostasis enables targeted protein degradation.

Despite the tremendous promise of PROTACs, their widespread application has been tempered by concerns of off-target effects and related toxicities.^6^ Protein degraders have been developed against a variety of clinically relevant protein targets in cancer, such as the androgen receptor^7^ and estrogen receptor,^8^ with candidates in clinical trials. However, the lack of precise control over their activity has raised concerns.^9^ The powerful catalytic nature of PROTACs may lead to uncontrolled degradation of the POIs in both pathological and healthy tissues, leading to broad-scale toxicity. For example, inhibition of BET bromodomains is relatively well tolerated, while complete loss of BRD2 and BRD4 is lethal, which reduces the therapeutic window of dBET1, a PROTAC targeting BET bromodomains.^10,11^ Moreover, the inherent promiscuity of traditional chemistries employed in PROTAC design can result in unintended interactions, leading to unwanted degradation of nontarget proteins and contributing to systemic toxicity.^12^ As such, there is an urgent need for advanced strategies to fine-tune the activity of PROTACs, ensuring controlled activation and avoiding collateral damage to the proteome.

To achieve precise control over the PROTAC activity, various stimulus-responsive strategies have been explored. The majority of these stimulus-responsive PROTACs persist in an inactive state until activated by endogenous or exogenous stimulus—turned “on” in specific tissues while remaining “silent” elsewhere. For example, in-cell click-formed proteolysis-targeting chimeras (CLIPTACs) are formed intracellularly through a click chemistry reaction between two PROTAC warheads, delivered separately.^13^ However, the inconsistent pharmacokinetic profiles of the two precursors and the inefficient in situ generation of the PROTAC are key limitations for clinical translation. It has also been demonstrated that PROTACs can be activated using endogenous stimuli, such as hypoxia^14,15^ or reactive oxygen species (ROS)^16^-activated moieties. However, the drawback of using internal stimuli, such as pH or redox potential, is that they are often located at multiple sites within the body resulting in an unintended leaky “off” state. Photocages have been installed onto PROTACs to switch “on” their activity with light.^17^ However, the therapeutic potential of light-activated PROTACs is limited by poor tissue penetration of light. In contrast to activation strategies, to our knowledge, there is only one method to switch “off” PROTAC activity—a crucial approach to minimizing off-target effects.^18^ The incorporation of photoswitches allows for reversible activation and deactivation of protein degradation activity upon UV/visible-light exposure. However, like the other light-dependent approaches, its therapeutic potential remains limited by poor tissue penetration of light. Thus, no clinically viable PROTAC “off” switch has been developed—an important feature for achieving temporal control and minimizing off-target activity.

In this study, we employ DNA nanotechnology to turn “off” PROTAC activity. DNA nanotechnology is ideal for this application due to its remarkable programmability and structural versatility. Guided by well-defined design principles, a wide array of DNA-based building blocks have been leveraged to create diverse, chemically tunable, and multidimensional architectures.^19^ Such DNA nanostructures show immense promise for biomedical applications ranging from targeted drug delivery^20^ to biosensing and molecular diagnostics.^21,22^ Moreover, the development of chemical modifications for nuclease stability and efficient delivery agents has accelerated the advancement of a new generation of nucleic acid therapeutics, with over 23 drugs on the market.^23^ Recently, efforts to incorporate DNA-based modules into PROTACs have opened new avenues for programmability and target selectivity. For instance, E3 ligase ligands have been conjugated to protein-specific oligonucleotides^24^ such as aptamers^25^ and double-stranded transcription factor-binding sequences,^26^ enabling selective degradation of nucleic acid-binding proteins. In parallel, DNA nanotechnology has been explored as a tool to control PROTAC functionality but with limited success. PROTAC warheads have been attached to large DNA tetrahedra for protein degradation, but these are expensive to synthesize, difficult to produce on a large scale, and static in nature.^27^ Hybridization chain reactions have also been used to assemble PROTACs in cells, but this requires complicated delivery procedures involving two separate DNA strands.^28^ Here, we have generated oligonucleotide-linked PROTACS (OligoPROTACs) through simple hybridization of two short DNA strands: one 3′-modified strand with a POI binder and the other 5′-modified with an E3 ligase binder. Introducing different numbers of noncomplementary base pair arms on the double-stranded DNA (dsDNA) linker allowed us to optimize the spatial positioning of the PROTAC warheads, to achieve optimal protein degradation. Through the incorporation of a toehold sequence into the OligoPROTAC, we have demonstrated a dynamic off-switch mechanism for controlling protein degradation activity. This was achieved by incubation with a third inhibitor strand, which disrupts the dsDNA duplex through a toehold-mediated strand displacement. This disruption of the duplex results in the separation of the PROTAC warheads, causing inactivation. Since the “off” switch is encoded in the DNA sequence, this approach offers a general mechanism for the precise regulation of the PROTAC function.

Our design of OligoPROTACs also favors direct clinical translation, as they are short, double-stranded, and chemically modified oligonucleotides similar to small interfering RNA (siRNA), which have already been translated to the clinic for multiple indications. Incorporation of the DNA nanotechnology capabilities via the simple toehold design will enable the enhancement of the safety and efficacy of PROTAC-based therapies. This study lays the groundwork for integrating DNA nanotechnology into the next generation of targeted protein degradation approaches, allowing for the design of “smart” therapeutics.

## Results

The small-molecule PROTAC AT1 ^29^ was chosen as the guide for OligoPROTAC design. AT1 shows a selective BET bromodomain protein, BRD4 degradation, and suppression of tumor growth in AML.^30^ AT1 comprises of the widely used and well-characterized binder of BET bromodomain proteins, (+)-JQ1,^31^ as its POI-targeting warhead, and the VH032 ligand, for recruiting the VHL E3 ligase complex.^32^ To create the OligoPROTAC, we aimed to attach VH032 to the 5′-terminus of one DNA oligonucleotide and (+)-JQ1 to the 3′-terminus of a complementary DNA oligonucleotide. Thus, by hybridizing the two DNA strands, the VH032 and (+)-JQ1 PROTAC warheads would be positioned in close proximity on the same end of the double strand.

To use a dsDNA sequence that would not be biologically active itself, we used a previously developed nontargeting control ASO sequence and its complement.^33^ We chose to modify all DNA strands with phosphorothioate (PS) backbones in order to ensure nuclease stability within cells. The 5′-VH032 OligoPROTAC strand was synthesized from a commercial 5′-hexynyl-modified PS DNA with a commercially sourced VH032-azide, through copper-catalyzed click chemistry (Figures 1a, S2, S4, S6, S8, S10, and S12). In a similar fashion, the 3′-(+)-JQ1 OligoPROTAC strand was synthesized from a commercial 3′-azide-modified PS DNA with (+)-JQ1 alkyne, through copper-catalyzed click chemistry (Figures 1b and S1, S3, S5, S7, S9, S11, S13, and S19). The (+)-JQ1 alkyne was synthesized in two steps: first, the Boc group of (+)-JQ1 was deprotected to yield the (+)-JQ1 acid;^34^ followed by a hydroxybenzotriazole (HoBT) and (3-Dimethylamino-propyl)-ethyl-carbodiimide hydrochloride (EDC-HCl)-mediated coupling with propargylamine, resulting in the formation of (+)-JQ1-alkyne.^35^ Both modified-DNA strands were purified using HPLC and analyzed by LC–MS, with yields >90% and purity >95%. The two strands were subsequently annealed to assemble OligoPROTAC (Figure 1c).

**Figure 1:**
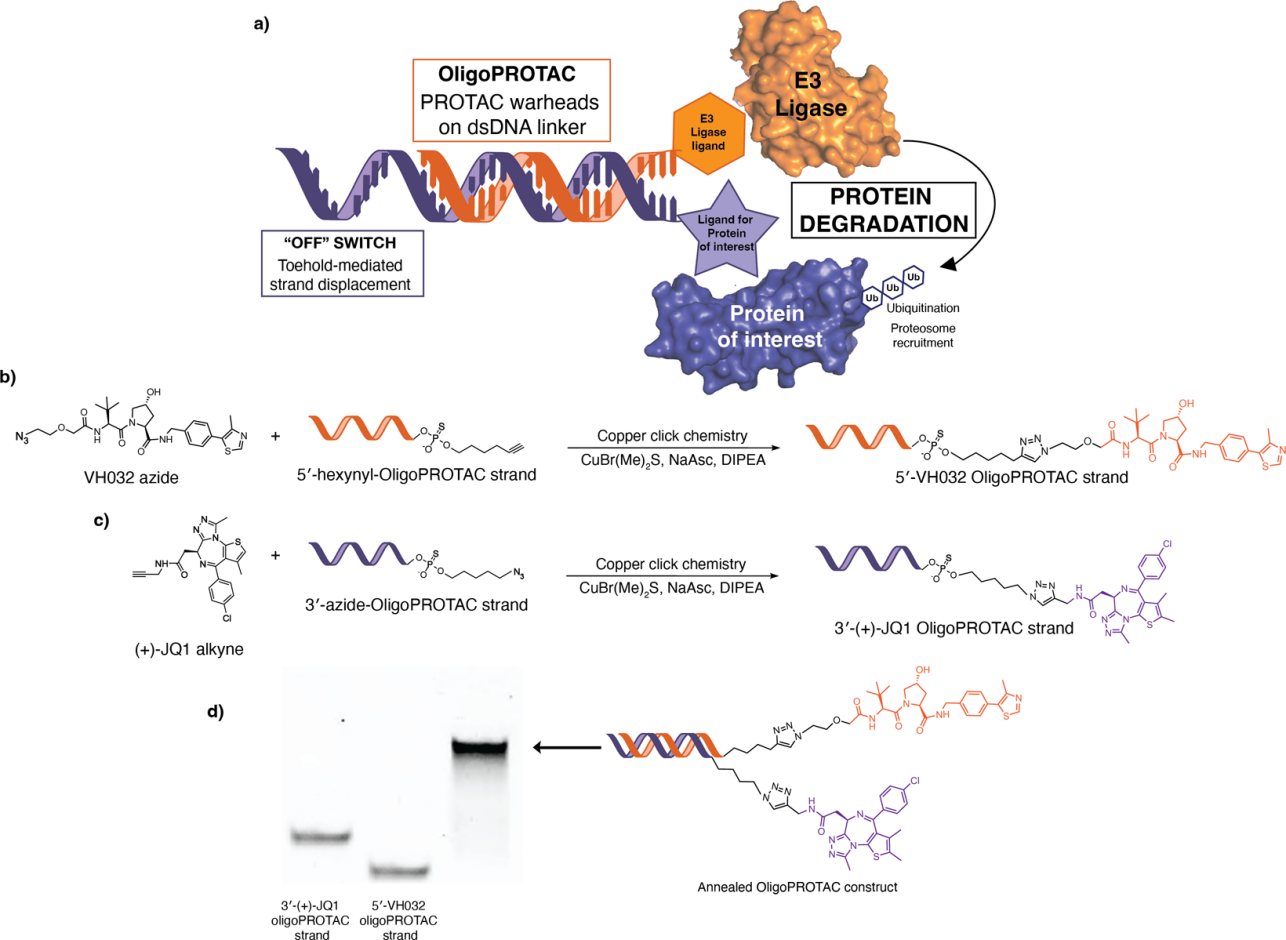
Chemical synthesis of OligoPROTACs. (a) OligoPROTACs utilize a double-stranded DNA linker to present PROTAC warheads, which recruit an E3 ligase to induce ubiquitination and subsequent degradation of a target protein. The system includes a toehold sequence to facilitate a toehold-mediated strand displacement mechanism as an “off” switch for controlled activation. (b) Reaction scheme for copper click conjugation of the VH032 azide with 5′-alkyne-modified PS DNA. (c) Reaction scheme for copper click conjugation of (+)-JQ1 alkyne with 3′-azide-modified PS DNA. (d) Native PAGE gel showing the assembly of dsDNA OligoPROTAC.

Since it is widely accepted that linker length plays a critical role upon the efficacy of small-molecule PROTACs, we chose to optimize linker lengths in our OligoPROTAC construct in an analogous manner. To vary the linker length and thus the spatial positioning of the VH032 and (+)-JQ1 ligands, we introduced additional noncomplementary thymine bases [*n* = 0, 1, 2, 3, 5, 10] between the ligands and the dsDNA region. These additional sequence “arms” increase the spatial separation between the ligands attached at each terminus (Figure 2a).

**Figure 2:**
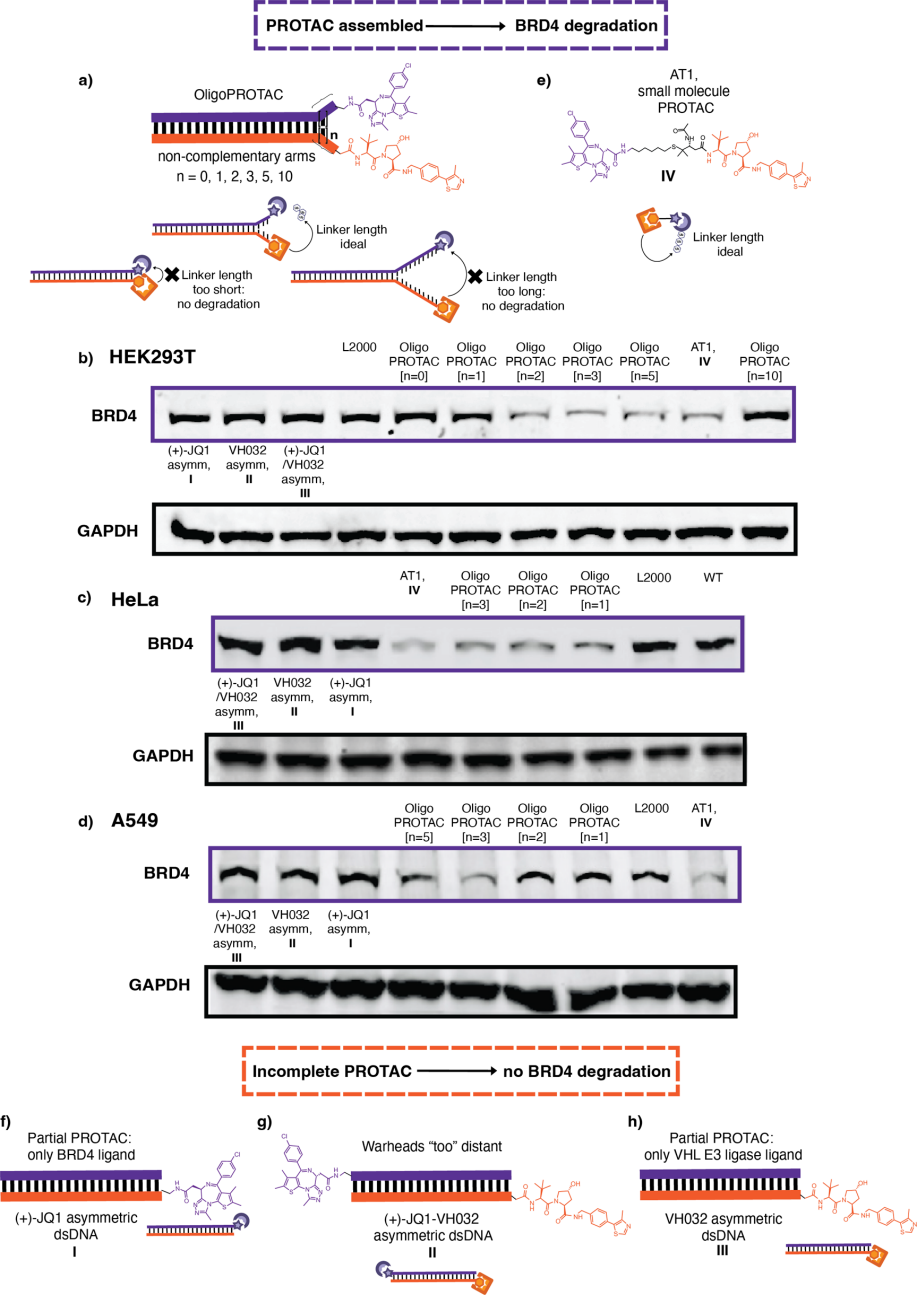
Screening of the OligoPROTAC dsDNA linker length. (a) Assembled dsDNA OligoPROTAC with noncomplementary thymine arms of varying lengths [*n* = 0, 1, 2, 3, 5, 10]. (b) Western blot for BRD4 levels following treatment with OligoPROTACs of varying dsDNA linker lengths, upon Lipofectamine 2000 transfection in HEK293T cells at 12 h. Normalized to GAPDH levels. (c) Western blot for BRD4 levels following treatment with OligoPROTACs of varying dsDNA linker lengths, upon Lipofectamine 2000 transfection in HeLa cells at 12 h. Normalized to GAPDH levels. (d) Western blot for BRD4 levels following treatment with OligoPROTACs of varying dsDNA linker lengths, upon Lipofectamine 2000 transfection in A549 cells at 12 h. Normalized to GAPDH levels. (e) Chemical structure of small-molecule PROTAC, AT1. (f) Structure for the (+)-JQ1 partial construct comprising of the 3′-(+)-JQ1 OligoPROTAC strand annealed to a nonmodified complementary strand. (g) Structure for the VH032 partial construct comprising of the 3′-(+)-JQ1 OligoPROTAC strand annealed to a nonmodified complementary strand. (h) Structure for the (+)-JQ1/VH032 asymmetric construct, the asymmetric oligoPROTAC, where the ligands are on opposite ends of the dsDNA (5′-VH032 and 5′-(+)-JQ1).

These OligoPROTAC designs, with varying lengths of noncomplementary arms, were transfected into HEK293T cells, and the resultant BRD4 degradation was measured by Western blotting at 12 h. We observed a distance-dependent degradation of the protein target, BRD4 (Figures 2b and S14, S17). OligoPROTAC constructs with no arms [*n* = 0] exhibited minimal degradation, with degradation efficiency increasing upon the introduction of noncomplementary thymine arms, up until *n* = 3. The degradation observed for *n* = 3 arms was comparable to that observed from the original small-molecule PROTAC AT1 (Figures 2b,e and S14, S17). Activity was reduced when increasing the length of the noncomplementary arms further [*n* = 5], due to the ligands being positioned too far apart. With *n* = 10 arms, there was no observable degradation (Figures 2b and S14, S18). We then extended our OligoPROTAC activity screening to HeLa (Figures 2c and S15) and A549 (Figures 2d and 16) cells. These lines were chosen to assess performance in potential target cancerous environments. As with the previous results, we also observed a similar distance-dependent BRD4 degradation, where *n* = 3 arms produced the highest level of protein degradation.

To confirm that protein degradation was dependent on the precise spatial arrangement of the ligands, we designed a series of control DNA constructs. Partial OligoPROTACs were generated by annealing the 3′-(+)-JQ1 OligoPROTAC strand with a nonmodified complementary strand (Figure 2f) and the 5′-VH032 OligoPROTAC strand with a nonmodified complementary strand (Figure 2g). We also constructed an asymmetric OligoPROTAC, where the ligands were on opposite ends of the dsDNA (5′-VH032 and 5′-(+)-JQ1) (Figures 2h and S13). None of these control constructs were able to elicit BRD4 degradation in any of the three cell lines, thereby confirming that the activity, i.e., BRD4 degradation, we observed was exclusively due to the OligoPROTAC holding the two PROTAC warheads in the optimal spatial configuration (Figures 2b,d and S14–S18).

We then benchmarked the best performer, OligoPROTAC [*n* = 3] against the small-molecule PROTAC AT1 to assess its protein degradation efficacy and gain insight into its mechanism of action. To determine the impact of OligoPROTAC concentration on BRD4 degradation, the activity of the two molecules was tested over a 12 h treatment period. Across all concentrations tested, the OligoPROTAC [*n* = 3] exhibited BRD4 degradation levels comparable to those of AT1 (Figures 3a and S19). Additionally, a time-course experiment at the highest tested concentration (1 μM) further confirmed that both compounds achieved a similar degradation efficiency with time (Figures 3b and S20). Furthermore, MYC protein and RNA levels were affected, as expected for BRD4-targeting PROTACs (Figures 3c,d and S21). We also observed phenotypic changes such as rounded morphology and reduced confluency (Figure 3e) indicative of cytotoxicity (Figures 3f and S22), assessed by CellTiter Glo, at levels similar to those induced by the small-molecule PROTAC. We then sought to visualize OligoPROTAC [*n* = 3] in its tertiary complex with BRD4 and VHL using the ICM-Pro modeling approach. The model was constructed using the crystal structure of MZ1 as a template (5T35 ^36^). MZ1 contains the same PROTAC warheads as AT1 but differs in linker composition (Figure S23).

**Figure 3:**
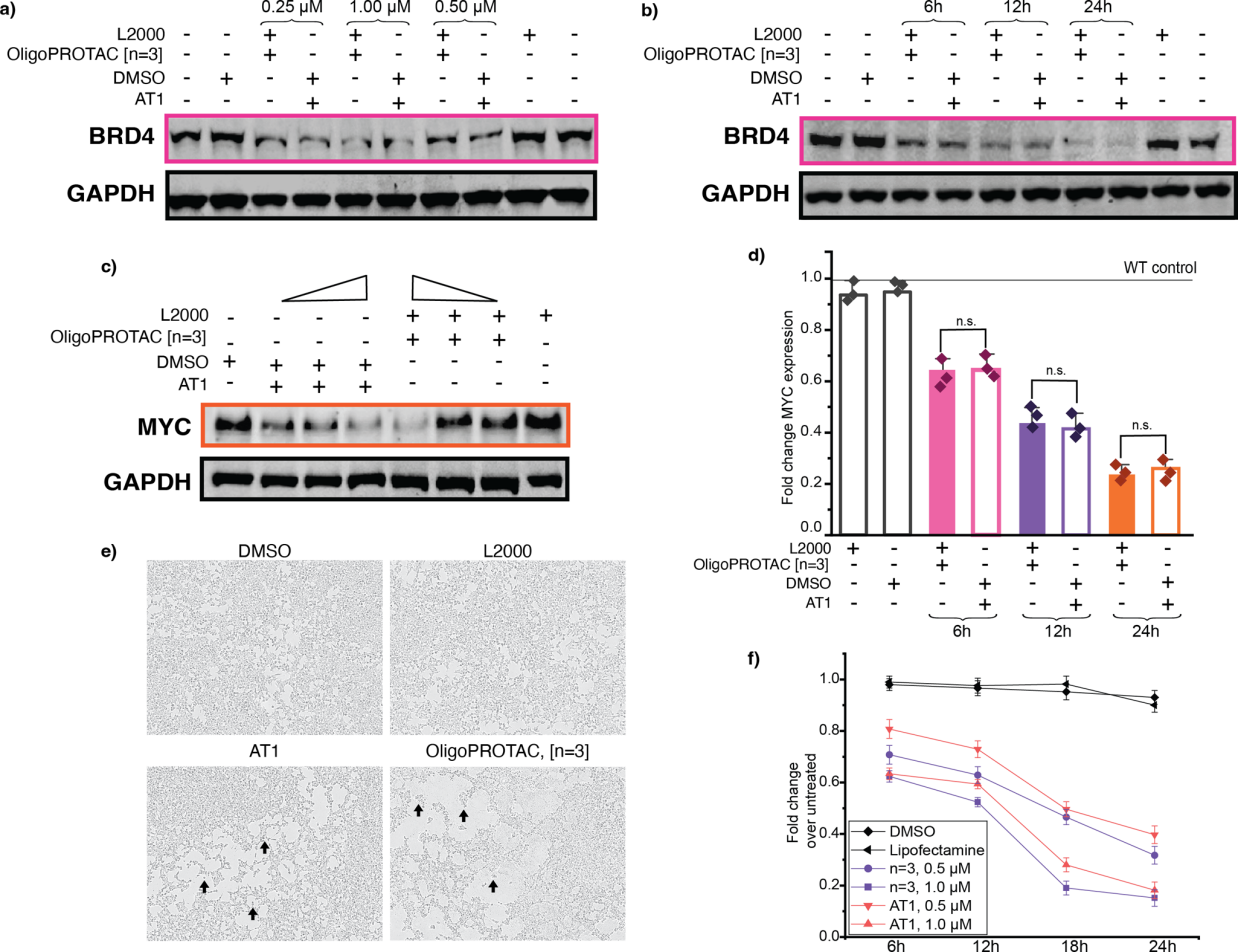
Evaluation of efficacy and mechanistic characterization of OligoPROTAC [*n* = 3] compared to the small molecule, AT1. (a) Western blot for BRD4 levels upon treatment with OligoPROTAC [*n* = 3] or small-molecule PROTAC, AT1, at concentrations indicated, upon Lipofectamine 2000 transfection/DMSO treatment in HEK293T cells at 12 h. Normalized to GAPDH levels. (b) Western blot for BRD4 levels upon treatment with OligoPROTAC [*n* = 3] or small-molecule PROTAC, AT1, at 1 μM over 6, 12, and 24 h, upon Lipofectamine 2000 transfection/DMSO treatment in HEK293T cells. Normalized to GAPDH levels. (c) Western blot for MYC levels upon treatment with OligoPROTAC [*n* = 3] or small-molecule PROTAC, AT1, at concentrations (0.25, 0.50, and 1.00 μM) upon Lipofectamine 2000 transfection/DMSO treatment in HEK293T cells at 12 h. Normalized to GAPDH levels. (d) RT-qPCR data for MYC expression upon lipofectamine transfection of OligoPROTAC [*n* = 3] or AT1 incubation in HEK293T cells at 1.00 μM over 6, 12, and 24 h. (e) Phase contrast images captured on the IncuCyte of HEK293T cells treated with OligoPROTAC [*n* = 3] or small-molecule PROTAC at 1.00 μM for 12 h. Arrows indicate cells with rounded morphology. (f) Cell viability upon treatment with OligoPROTAC [*n* = 3] or small-molecule PROTAC at a concentration indicated over a 24 h time period assessed by CellTiter Glo.

Thus, the OligoPROTACS show a comparable degradation efficacy to small-molecule PROTACs in terms of the dose–response profile and BRD4 degradation kinetics. Moreover, the mechanistic similarities such as downstream molecular targets and cytotoxicity of the OligoPROTAC and small-molecule PROTAC highlight that the DNA linker provides a unique opportunity to control activity without affecting the efficacy or mechanism.

To leverage the presence of a DNA linker, we aimed to use toehold-mediated strand displacement, a reaction widely employed in DNA nanotechnology, to turn “off” the OligoPROTAC (Figure 4a). For this, we chose to continue with the OligoPROTAC [*n* = 3] noncomplementary thymine arms as it possessed the highest degradation efficacy. Initially, we tested 5′ toehold sequences of the 3′-(+)-JQ1 OligoPROTAC strand (without the (+)-JQ1) ranging in length from 7 to 10 bases (Figure S24), based on literature sequences.^37^ These were annealed to the previous 5′-VH042 OligoPROTAC strand (without the VH032), which produced a dsDNA construct with 7–10 base -long ssDNA toeholds on one end, and the [*n* = 3] arms at the other end. As observed by gel electrophoresis, a fully complementary DNA strand to the extended toehold 3′-(+)-JQ1 OligoPROTAC strand (antitoehold complement) initiated toehold-mediated strand displacement and displaced the 5′-VH032 OligoPROTAC strand in all cases. An analogous reaction in cells should stop the activity of the OligoPROTAC, by separating the (+)-JQ1 and VH032 in space. As all sequences worked, we picked the 8-base toehold for future use, as it had been previously shown to function optimally within cells.^31^

**Figure 4:**
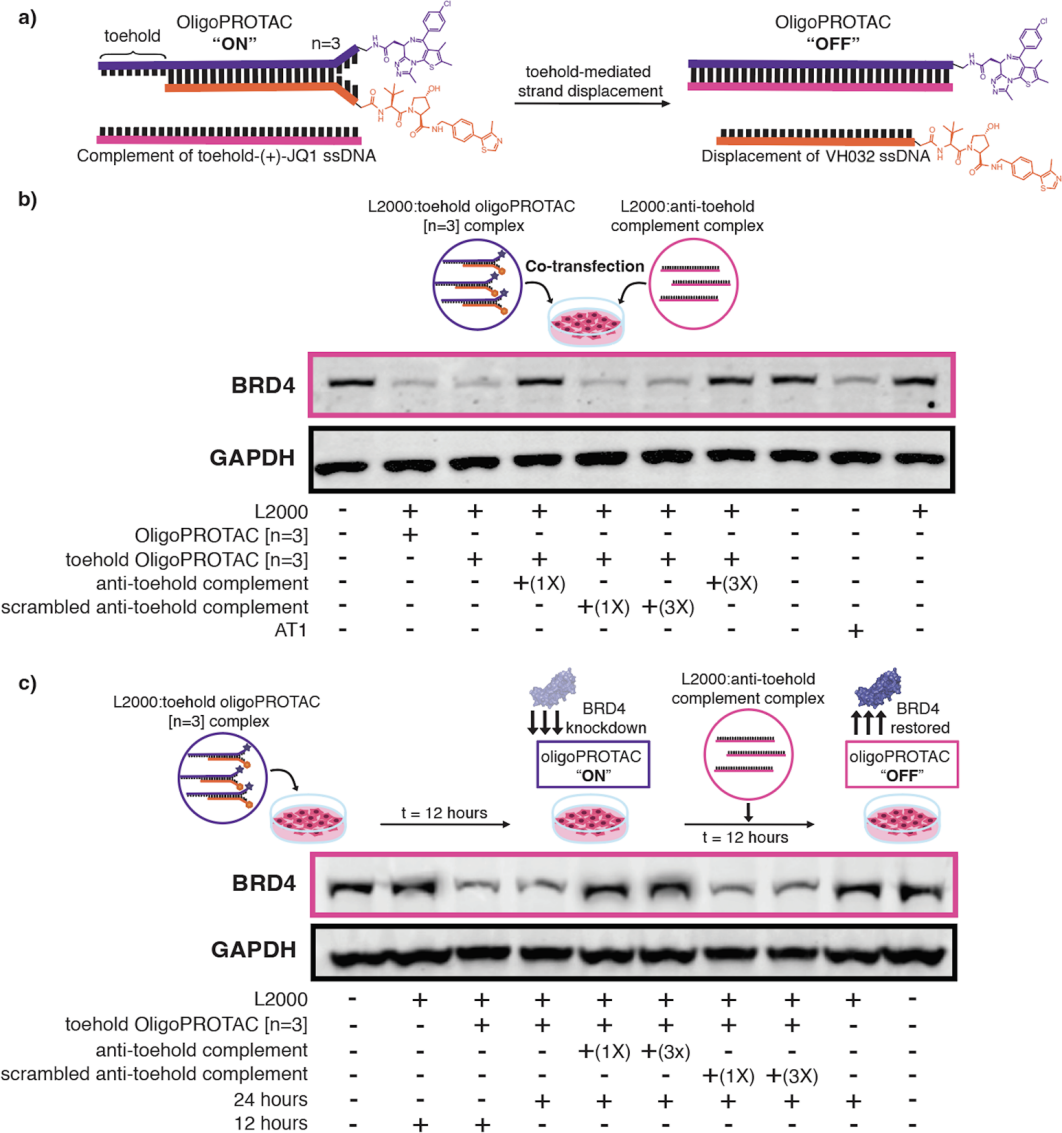
Dynamic “off” switch introduced into the OligoPROTAC through toehold-mediated strand displacement. (a) Schematic for the toehold-mediated strand displacement reaction between the toehold OligoPROTAC [*n* = 3] with the antitoehold complement, resulting in disruption of the dsDNA OligoPROTAC duplex and loss of activity. (b) Western blot of BRD4 levels upon cotransfection with toehold OligoPROTAC [*n* = 3], along with the antitoehold complement and scrambled antitoehold complement. Separate Lipofectamine 2000 nanoparticles were produced for the OligoPROTAC and antitoehold sequences, prior to transfection in HEK293T cells. Normalized to GAPDH levels. (c) Western blot of BRD4 levels upon treatment with toehold OligoPROTAC [*n* = 3] for 12 h, followed by the antitoehold complement and scrambled antitoehold complement and harvesting at 24 h. HEK293T cells were treated with the toehold OligoPROTAC [*n* = 3] for 12 h and activity was then turned “off” with a second transfection with the antitoehold complement. Normalized to GAPDH levels.

To test our “off” switch in cells, we synthesized the new extended 3′-(+)-JQ1 OligoPROTAC strand containing an 8-base toehold sequence at the 5′-end (Figure S25). This was annealed to the previous 5′-VH032 OligoPROTAC strand, which produced a dsDNA construct with 8 bases of ssDNA on one end (8-base toehold), and the (+)-JQ1 and VH032 ligands on *n* = 3 arms at the other end. This toehold OligoPROTAC [*n* = 3] was transfected into HEK293T cells and resultant BRD4 degradation was measured by Western blotting at 12 h. As expected, the 8-base toehold made no difference to the efficacy of BRD4 degradation with the toehold OligoPROTAC [*n* = 3], giving comparable efficiency to the previous OligoPROTAC [*n* = 3]. To turn off the BRD4 degradation in cells, separate lipid nanoparticles containing the Lipofectamine 2000/toehold OligoPROTAC [*n* = 3] and Lipofectamine 2000/antitoehold complement strand were cotransfected into HEK293T cells. This was to ensure that the toehold-mediated strand displacement would occur only inside the cell, following successful transfection of both nanoparticle species. We attempted the cotransfection with 1:1 and 1:3 ratios for the toehold OligoPROTAC [*n* = 3]:antitoehold complement. Excitingly, upon cotransfection with the antitoehold complement strand, the degradation activity of the OligoPROTAC was lost (Figures 4b and S26). This was observed for both ratios of the antitoehold complement. By cotransfecting a scrambled version of the antitoehold complement strand (in the same ratios), protein degradation was still observed.

We then decided to test whether, following knockdown of BRD4 with the toehold OligoPROTAC [*n* = 3], toehold-mediated strand displacement could dynamically turn the OligoPROTAC “off” and restore BRD4 levels within the cells. To do this, we first transfected the HEK293T cells with the toehold OligoPROTAC [*n* = 3] for 12 h. We then transfected the antitoehold complement or the scrambled antitoehold complement for another 12 h and harvested protein samples at 24 h. Strikingly, the antitoehold complement was able to restore BRD4 levels, thereby turning “off” the OligoPROTAC (Figures 4c, S27, and S28). This effect was again sequence-specific, as the scrambled antitoehold complement showed no restoration of BRD4 protein levels and therefore no inhibition of the OligoPROTAC activity. These experiments demonstrated that the loss of OligoPROTAC activity, i.e., the “off” switch, was due to the sequence-specific toehold-mediated strand displacement reaction occurring within the cells. Thus, we demonstrate the first instance of a sequence-specific “off” switch for controlling the PROTAC activity.

## Discussion

PROTACs represent a promising class of targeted protein degradation technologies, but their clinical applications are limited by off-target effects and toxicity. Thus, there is a need to develop methods to fine-tune their activity. To address these challenges, we present OligoPROTACs: containing PROTAC warheads positioned in space by a short dsDNA linker. Our OligoPROTACs achieved proximity-dependent protein degradation, which can be turned “off” by simply adding a third inhibitor DNA strand. This inhibitor strand separates the two strands of the dsDNA linker and hence the two PROTAC warheads through toehold-mediated strand displacement. As this control mechanism does not depend on the warheads used, it can serve as a general mechanism to control the activity of other PROTACs or any class of heterobifunctional molecules.

This study highlights the potential of DNA-based systems to minimize off-target effects of PROTACs through a rational design. We have validated OligoPROTAC functionality across diverse cell lines—HEK293T, HeLa, and A549—representing different tissue types and biological contexts. This highlights the broad applicability and robustness of our technological platform for noncancerous and cancerous cellular environments. The programmability of DNA enables the precise tuning of OligoPROTAC structures, including the length, sequence, and binding affinities of the various components. While we only include phosphorothioates for nuclease stability, further chemical modifications can be included for increased stability and improved pharmacodynamic/pharmacokinetic properties. This is possible as we do not rely upon enzymatic recognition of the nucleic acid component for activity.^38,39^ Moreover, OligoPROTACs closely resemble clinically approved short, double-stranded nucleic acid drugs, such as siRNA, in both structure and chemical composition. Their double-stranded architecture and phosphorothioate-modified backbone make them amenable to a wide range of established siRNA delivery strategies, including lipid nanoparticle formulations and conjugation to targeting ligands such as lipids, sugars, peptides, or antibodies.^40^ While this study focuses on in vitro validation, the clinical success of siRNA therapeutics provides a strong precedent for the future in vivo development and translation of OligoPROTACs.

The implications of this work extend beyond improving the safety, efficacy, and clinical translation of PROTAC therapies. The integration of DNA nanotechnology into protein degradation strategies opens the door to a broader class of “smart” therapeutics capable of adaptive responses to biological cues. For example, OligoPROTACs could be engineered to respond to disease-specific biomarkers, ensuring that therapeutic activity is restricted to pathological conditions. Furthermore, the modularity of DNA nanotechnology provides opportunities for multiplexed targeting, enabling the simultaneous degradation of multiple proteins in a coordinated manner.

In conclusion, OligoPROTACs represent a significant step forward in the development of precision-protein degraders. By harnessing the programmability and versatility of DNA nanotechnology, this approach provides a new layer of control over therapeutic activity, paving the way for safer, “smarter”, and thus more effective protein degradation therapies.

## Supplementary Material



## Data Availability

All the data generated in this study are available within the article, the Supporting Information, and figures. Source data are available from https://doi.org/10.5281/zenodo.16266181.

## Notes

Detailed methods for all organic syntheses, oligonucleotide functionalization and purification, and biological experiments; full characterization data including NMR, HR-MS, and oligonucleotide HPLC and MS; oligonucleotide and qPCR primer sequences; synthetic routes for OligoPROTAC functionalization; analytical validation; and OligoPROTAC activity assays such as cytotoxicity, qPCR, and Western blotting (PDF)
