# Correction to “DNA-Programmable Protein Degradation: Dynamic Control of Proteolysis-Targeting Chimera Activity via DNA Hybridization and Strand Displacement”

**DOI:** 10.1021/jacsau.5c01700

**Published:** 2026-01-09

**Authors:** Disha Kashyap, Shozeb Haider, Thomas A. Milne, Michael J. Booth

None of the conclusions of the paper have changed. However, a colleague initially spotted a mismatch in a pair of Western blot gels in our Supporting Information, and after this we initiated a full check of all raw data files. Following this, we have identified a few minor errors that require correction in both the main article and the Supporting Information. Corrections to the manuscript are given below, including a listing of changes applied to the Supporting Information file.

## Corrections to Main Text

Figure 1: The (+)-JQ1 molecule in Figure 1c was missing a methyl group, this has been corrected.

**Figure 1:**
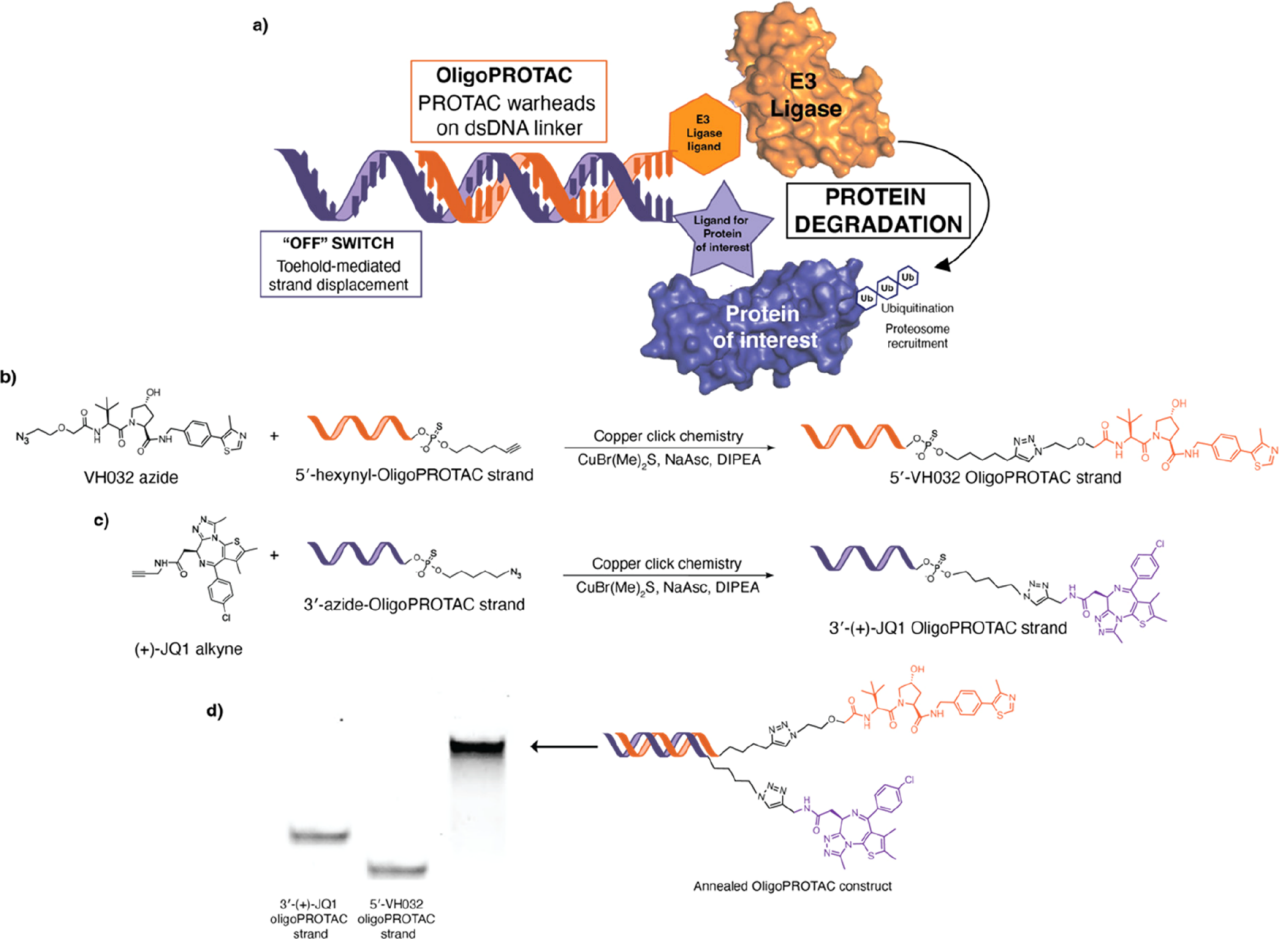
Chemical synthesis of oligoPROTACs. a) OligoPROTACs utilize a double-stranded DNA linker to present PROTAC warheads, which recruit an E3 ligase to induce ubiquitination and subsequent degradation of a target protein. The system includes a toehold sequence to facilitate a toehold-mediated strand displacement mechanism as an “off” switch for controlled activation. b) Reaction scheme for copper click conjugation of VH032 azide with 5′-alkyne modified PS DNA. c) Reaction scheme for copper click conjugation of (+)-JQ1 alkyne with 3′-azide modified PS DNA. d) Native PAGE gel showing assembly of dsDNA oligoPROTAC.

Figure 3: The GAPDH blot in Figure 3b has been updated to the correct image. We went back to the original raw data files to check this.

**Figure 3:**
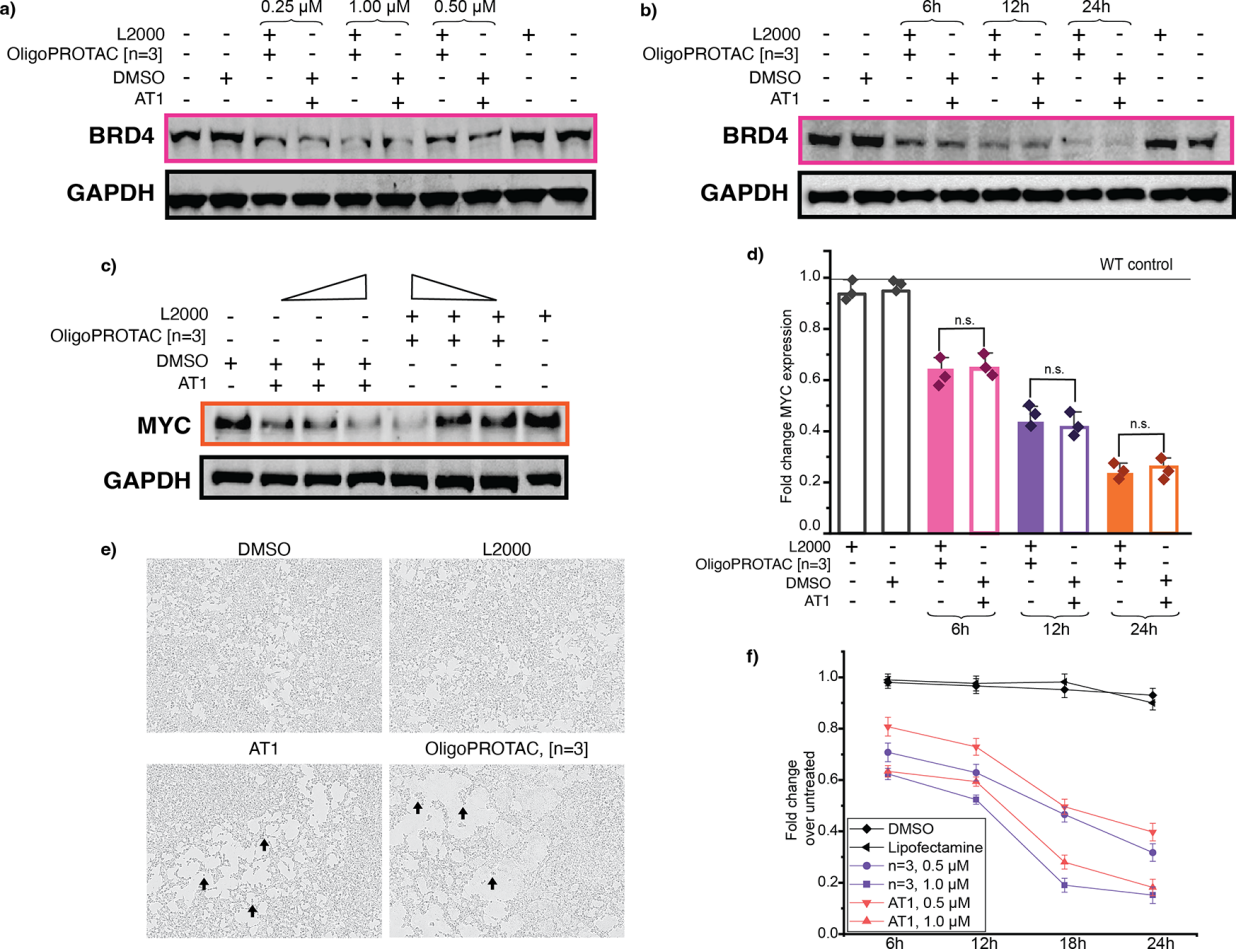
Evaluation of efficacy and mechanistic characterization of oligoPROTAC [*n* = 3] compared to small molecule, AT1. a) Western blot for BRD4 levels upon treatment with oligoPROTAC [*n* = 3] or small molecule PROTAC, AT1, at concentrations indicated, upon Lipofectamine 2000 transfection/DMSO treatment in HEK293T cells at 12 h. Normalized to GAPDH levels. b) Western blot for BRD4 levels upon treatment with oligoPROTAC [*n* = 3] or small molecule PROTAC, AT1, at 1 μM over 6, 12, and 24 h, upon Lipofectamine 2000 transfection/DMSO treatment in HEK293T cells. Normalized to GAPDH levels. c) Western blot for MYC levels upon treatment with oligoPROTAC [*n* = 3] or small molecule PROTAC, AT1, at concentrations (0.25, 0.50, and 1.00 μM) upon Lipofectamine 2000 transfection/DMSO treatment in HEK293T cells at 12 h. Normalized to GAPDH levels. d) RT-qPCR data for MYC expression upon lipofectamine transfection of oligoPROTAC [*n* = 3] or AT1 incubation in HEK293T cells at 1.00 μM over 6, 12, and 24 h. e) Phase contrast images captured on the Incucyte of HEK293T cells treated with oligoPROTAC [*n* = 3] or small molecule PROTAC at 1.00 μM for 12 h. Arrows indicate cells with rounded morphology. f) Cell viability upon treatment with oligoPROTAC [*n* = 3] or small molecule PROTAC at concentration indicated over a 24-h time period assessed by CellTiter Glo.

## Corrections to Supporting Information

In the section “Nucleic acid chemistry functionalization, purification, and characterization”, we have added additional information regarding what solvent the stock solution was made in for example: “To a 0.5 mL Eppendorf DNA LoBind tube was added, listed in order of addition, 1 μL of the DNA (1 mM stock concentration in KPhos pH 7.4), 1.5 μL of 1 mM JQ1-alkyne (stock in DMF), molecule **2**, 1 μL of 200 mM DIPEA, 4.5 μL of H_2_O, 1 μL of 200 mM sodium ascorbate and finally, 1 μL of 200 mM CuBrMe_2_S (stock in DMSO). The reaction was vortexed, spun down in a tabletop centrifuge and placed in a Thermomixer (Eppendorf) overnight, shaking at 800 rpm at room temperature.”

Under the section “Alternate fully aqueous reaction conditions for copper click chemistry”, we have also added another reaction procedure for fully aqueous copper click chemistry used in the lab. Some conjugates were also prepared using this method and used for initial linker optimization studies; however, for all the main text data we used CuBrMe_2_S/DIPEA chemistry.

Supplementary Figure 15: The GAPDH blot has been flipped 180° to reflect correct orientation.

Supplementary Figure 16: The cell type in the legend has been corrected to reflect the BRD4 levels in cell line A549.

Supplementary Figure 17: The BRD4 blot had been cropped incorrectly and was missing the L2000 lane at the right end; this has now been fixed.

Supplementary Figure 20: The GAPDH blot has switched for the correct image. We went back to the original raw data files to check this.

Supplementary Figure 26: Lane annotations were incorrect; this has now been fixed.

Supplementary Figure 28: Lane annotations were incorrect; this has now been fixed.

## Supplementary Material



## Notes

Detailed methods for all organic syntheses, oligonucleotide functionalization and purification, and biological experiments; full characterization data including NMR, HR-MS, oligonucleotide HPLC and MS; oligonucleotide and qPCR primers sequences. Figures S1–S28 contain synthetic routes for OligoPROTAC functionalization, analytical validation, OligoPROTAC activity assays such as cytotoxicity, qPCR and Western blotting (PDF)

