# Supplementary material for: Correction to “DNA-Programmable Protein Degradation: Dynamic Control of Proteolysis-Targeting Chimera Activity via DNA Hybridization and Strand Displacement”
Source: JACS Au. 2026 Jan 9;6(1):653–5. doi: 10.1021/jacsau.5c01700 (PMC12848738; doi:10.1021/jacsau.5c01700)
Supplement: Supplementary file 1 [file au5c01700_si_001.pdf]

Supplementary Information for:

DNA-programmable Protein Degradation: Dynamic Control of PROTAC Activity via DNA Hybridization and Strand Displacement

Disha Kashyap[1,2], Shozeb Haider[3,4,5], Thomas A. Milne[2,*], Michael J. Booth[1,6,*]

[1]Department of Chemistry, University of Oxford, Mansfield Road, Oxford, OX1 3TA, U.K.
[2]MRC Molecular Haematology Unit, MRC Weatherall Institute of Molecular Medicine, Radcliffe Department of Medicine, University of Oxford, Oxford, OX3 9DS, U.K.
[3]UCL School of Pharmacy, University College London, London WC1N 1AX, U.K.
[4]University of Tabuk (PFSCBR), Tabuk, 71491, Saudi Arabia
[5]UCL Centre for Advanced Research Computing, University College London, WC1H 9RL, U.K.
[6]Department of Chemistry, University College London, 20 Gordon Street, London, WC1H 0AJ, U.K.

*Correspondence: thomas.milne@imm.ox.ac.uk, m.j.booth@ucl.ac.uk

Table of Contents

1. **Materials and Methods**……………………………………………………………………………….........2
   1.1. Organic Synthesis
      1.1.1. General information about reagents, purification, and characterisation
      1.1.2. (S)-2-(4-(4-chlorophenyl)-2,3,9-trimethyl-6H-thieno[3,2-f][1,2,4]triazolo[4,3-a][1,4]diazepin-6-yl)acetic acid
      1.1.3. (S)-2-(4-(4-chlorophenyl)-2,3,9-trimethyl-6H-thieno[3,2-f][1,2,4]triazolo[4,3-a][1,4]diazepin-6-yl)-N-(prop-2-yn-1-yl)acetamide
   1.2. Nucleic Acid Chemistry Functionalisation, Purification, and Characterisation
      1.2.1. (+)-JQ1-functionalisation with copper click chemistry
      1.2.2. VH032-functionalisation with copper click chemistry
      1.2.3. Assembly of OligoPROTAC
      1.2.4. Oligonucleotide MS characterisation

2. **Nucleic Acid Sequences**…………………………………………………………………………………….4
   2.1. Table 1: OligoPROTAC and toehold-sequences used
   2.2. Table 2: Molecular weights for Oligonucleotide conjugates prepared

3. **Biological Assays**…………………………………………………………………………….….........5
   3.1. Cell Culture
   3.2. Transfection
   3.3. Western Blotting
   3.4 Molecular Modelling

4. **Supplementary Figures**…………………………………………………………………………..………..7
   4.1. Figure S1: Reaction and characterisation for (+)-JQ1 ssDNA OligoPROTAC, [n=0]
   4.2. Figure S2: Reaction and characterisation for VH032 ssDNA OligoPROTAC, [n=0]
   4.3. Figure S3: Reaction and characterisation for (+)-JQ1 ssDNA OligoPROTAC, [n=1]
   4.4. Figure S4: Reaction and characterisation for VH032 ssDNA OligoPROTAC, [n=1]
   4.5. Figure S5: Reaction and characterisation for (+)-JQ1 ssDNA OligoPROTAC, [n=2]
   4.6. Figure S6: Reaction and characterisation for VH032 ssDNA OligoPROTAC, [n=2]
   4.7. Figure S7: Reaction and characterisation for (+)-JQ1 ssDNA OligoPROTAC, [n=3]
   4.8. Figure S8: Reaction and characterisation for VH032 ssDNA OligoPROTAC, [n=3]
   4.9. Figure S9: Reaction and characterisation for (+)-JQ1 ssDNA OligoPROTAC, [n=5]
   4.10. Figure S10: Reaction and characterisation for VH032 ssDNA OligoPROTAC, [n=5]
   4.11. Figure S11: Reaction and characterisation for (+)-JQ1 ssDNA OligoPROTAC, [n=10]
   4.12. Figure S12: Reaction and characterisation for VH032 ssDNA OligoPROTAC, [n=10]
   4.13 Figure S13: Reaction and characterisation for (+)-JQ1 asymmetric ssDNA OligoPROTAC, [n=3]
   4.14 Figure S14: Uncropped western blot of BRD4 levels upon lipofectamine 2000 transfection in HEK293T with OligoPROTAC of varying linker lengths and partial PROTAC control constructs

4.15 Figure S15: Uncropped western blot of BRD4 levels upon lipofectamine 2000 transfection in HEK293T with OligoPROTAC of varying linker lengths, [n=1, 2, 3] and partial PROTAC control constructs
4.16 Figure S16: Uncropped western blot of BRD4 levels upon lipofectamine 2000 transfection in HEK293T with OligoPROTAC of varying linker lengths, [n=3, 5, 10] and control constructs
4.17 Figure S17: Uncropped western blot of BRD4 levels upon lipofectamine 2000 transfection in HeLa with OligoPROTAC of varying linker lengths and partial PROTAC control constructs
4.18 Figure S18: Uncropped western blot of BRD4 levels upon lipofectamine 2000 transfection in A549 with OligoPROTAC of varying linker lengths and partial PROTAC control constructs
4.19 Figure S19: Uncropped western blot for BRD4 levels upon treatment with n=3 OligoPROTAC and small molecule PROTAC, AT1 at concentrations indicated over upon Lipofectamine 2000 transfection/DMSO treatment in HEK293T cells at 12 hours.
4.20 Figure S20: Uncropped western blot for BRD4 levels upon treatment with n=3 OligoPROTAC and small molecule PROTAC, AT1 at 1.00 µM over 6 hours, 12 hours, and 24 hours upon Lipofectamine 2000 transfection/DMSO treatment in HEK293T cells
4.21 Figure S21: Uncropped western blot for MYC levels upon treatment with n=3 OligoPROTAC and small molecule PROTAC, AT1 at concentrations (0.25, 0.50, 1.00 µM) upon Lipofectamine 2000 transfection/DMSO treatment in HEK293T cells at 12 hours
4.22 Figure S22: Viability of the HEK293T upon treatment with OligoPROTAC of varying linker lengths and partial PROTAC control constructs evaluated by Cell-Titer Glo
4.23 Figure S23: Molecular model of OligoPROTAC, [n=3] compared to small molecular PROTAC, MZ1.
4.24 Figure S24: Native PAGE gel for screening toehold sequences for toehold mediated strand displacement "off" switch for OligoPROTAC construct
4.25 Figure S25: Reaction and characterisation for toehold-(+)-JQ1 ssDNA OligoPROTAC, [n=3]
4.26 Figure S26: Uncropped western blot of BRD4 levels upon treatment with toehold OligoPROTAC [n=3], along with anti-toehold complement and scrambled anti-toehold complement upon lipofectamine 2000 transfection in HEK293T cells
4.27 Figure S27: Uncropped western blot of BRD4 levels upon treatment with toehold OligoPROTAC [n=3] for 12 hours, followed by anti-toehold complement and scrambled anti-toehold complement incubation for another 12 hours – harvest at 24 hours
4.28 Figure S28: Uncropped western blot of BRD4 levels upon treatment with toehold OligoPROTAC [n=3] for 12 hours, followed by anti-toehold complement and scrambled anti-toehold complement incubation for another 12 hours – harvest at 24 hours (biological replicate)

5. **References**……………………………………………………………………………………………**20**

Materials and Methods

Organic synthesis

General information

Reagents were purchased from commercial sources (Merck, BroadPharm and Sigma-Aldrich) and used without further purification. Dry solvents were taken from a solvent drying system (MBraun MB-SPS-5-Bench Top) under nitrogen atmosphere (H_2O content < 20 ppm as determined by Karl Fischer titration). Eluent mixtures are reported in %vol and volume:volume. Column chromatography was carried out using an automated Biotage Selekt One purification machine with Biotage Sfär Silica or C18 Bio Duo flash chromatography cartridges. HPLC grade solvents were used for purifications, extractions, and workups. TLC was carried out on Merck silica gel 60 F254 Al plates visualized under UV light (254 nm) or by staining with permanganate.

NMR spectroscopy measurements were recorded using a Bruker AVII400 or AVIII600 instrument and peaks were referenced to the residual solvent peak. Mass Spectrometry (MS) measurements for small molecules synthesised was performed on an ACQUITY I-Class PLUS UPLC System (Waters, Milford, MA, USA) coupled to an ACQUITY RDa mass spectrometer (Waters, Milford, MA, USA) equipped with an ESI probe, in positive ion mode. An ACE Equivalence 3 C18 column (50 x 2.1 mm, 3 µm; Avantor, Radnor, PA, USA) at 40C, was used with mobile phase A: water + 0.1 % formic acid, and mobile phase B: acetonitrile + 0.1% formic acid. The linear gradient used was: 0 minutes, 5% B; 0.5 minutes, 5% B; 4.0 minutes 95% B; 4.25 minutes, 95% B; 4.26 minutes, 5% B; and 5 minutes, 5% B. The flow rate was 0.4 mL/minute and the total analysed time was 5 minutes.

1.**(S)-2-(4-(4-chlorophenyl)-2,3,9-trimethyl-6H-thieno[3,2-f][1,2,4]triazolo[4,3-a][1,4]diazepin-6-yl)acetic acid**

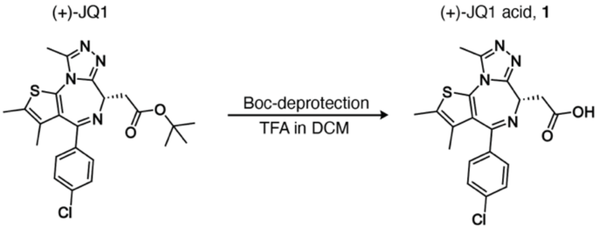

Following a literature procedure[1], to a stirred solution of (+)-JQ1 (90.0 mg, 0.2 mmol) in dichloromethane (4.0 mL) was added trifluoroacetic acid (0.5 mL). The reaction mixture was stirred at room temperature for 2 hours. The reaction mixture was concentrated under vacuum, and purified via a C18 reversed flash chromatography (5 to 100% MeOH : H_2O) to afford JQ1 carboxylic acid, **1** (78.0 mg, 99% yield) as a white solid. ^1H NMR (400 MHz, $CDCl_3$) δ 7.43 (d, J = 8.2 Hz, 2H), 7.33 (d, J = 8.2 Hz, 2H), 4.60 (t, J = 6.8 Hz, 1H), 3.72 (dd, 1H), 3.60 (dd, J = 16.8, 6.7 Hz, 1H), 2.69 (s, 3H), 2.41 (s, 3H), 1.69 (s, 3H). ^{13}C NMR (101 MHz, CDCl3) δ 173.25, 164.33, 137.13, 136.17, 131.94, 131.39, 131.06, 130.55, 130.00, 128.81, 53.64, 36.69, 14.45, 13.17, 11.77. (ESI, m/z): MS (ESI+) found m/z 401.0835 (M+H)+.

2.**(S)-2-(4-(4-chlorophenyl)-2,3,9-trimethyl-6H-thieno[3,2-f][1,2,4]triazolo[4,3-a][1,4]diazepin-6-yl)-N-(prop-2-yn-1-yl)acetamide**

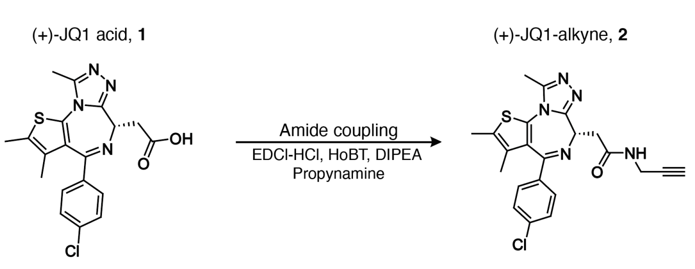

Following a patented procedure[2], to a solution of (+)-JQ1 acid, **1** (160 mg, 0.4 mmol), prop-2-yn-1-amine (26.4 mg; 0.5 mmol), EDCl-HCl (84.2 mg; 0.44 mmol), and HOBt (74.4 mmol; 0.44 mmol) in DMF (2 ml) was added DIPEA (181

mg; 1.4 mmol). The reaction mixture was stirred at room temperature overnight. The reaction mixture was subsequently, diluted with water and extracted with ethyl acetate. The organic layer was washed with brine, dried over Na_2SO_4, and concentrated. The resulting residue was purified by normal phase column chromatography with (0 to 10% MeOH : DCM) to give JQ1-alkyne, **2** (159 mg; 87.8 %) as an off-white oil. ^{1}H NMR (600 MHz, $CDCl_3$) δ 7.42 (d, J = 8.1 Hz, 2H), 7.32 (d, J = 8.6 Hz, 2H), 6.93 (br s, 1H), 4.63 (dd, J = 6.5 Hz, 6.5 Hz, 1H), 4.18 (ddd, J = 17.5 Hz, 5.6 Hz, 2.4 Hz, 1H), 3.97 (ddd, J = 17.5 Hz, 4.5 Hz, 2.3 Hz, 1H), 3.33 (dd, J =14.0 Hz, 6.5 Hz, 1H), 2.60 (s, 3H), 2.33 (s, 3H), 2.14 (t, J = 2.5 Hz, 1H), 1.60 (s, 3H). ^{13}C NMR (151 MHz, $CDCl_3$) δ 170.12, 155.32, 150.08, 137.06, 136.10, 131.63, 131.33, 131.01, 130.65, 130.1, 128.67, 79.38, 71.39, 54.09, 38.64, 29.28, 14.31, 13.05, 11.68. MS (ESI, m/z): MS (ESI+) found m/z 438.1141 (M+H)+.

Nucleic acid chemistry functionalisation, purification, and characterisation

(+)-JQ1-functionalisation with copper click chemistry

To a 0.5 mL Eppendorf DNA LoBind tube was added, listed in order of addition, 1 µL of the DNA (1 mM stock concentration in KPhos pH 7.4), 1.5 µL of 1 mM JQ1-alkyne (stock in DMF), molecule **2**, 1 uL of 200 mM DIPEA, 4.5 µL of H_2O, 1 µL of 200 mM sodium ascorbate and finally, 1 µL of 200 mM $CuBrMe_2S$ (stock in DMSO). The reaction was vortexed, spun down in a tabletop centrifuge and placed in a Thermomixer (Eppendorf) overnight, shaking at 800 rpm at room temperature.

The reaction was quenched with 250 µL of 0.5 M EDTA pH 8, made up to 500 µL with H_2O and first run through a Amicon 3K 0.5 mL column/tube as described above to chelate excess copper and remove it along with the unreacted small molecule. The final eluent collected after inversion was then purified by HPLC on an Agilent Polaris C18 column (150 x 4.6 mm), column heated to 50°C using a gradient of 3-30% or 3-50% CH_3CN (indicated in **SI** figures) over 20 minutes, flow rate of 1.5 mL/min, with 10 mM triethylammonium bicarbonate (TEAB) pH 8.5 as an ion-pairing buffer throughout.

VH032-functionalisation with copper click chemistry

To a 0.5 mL Eppendorf DNA LoBind tube was added, listed in order of addition, 1 µL of the DNA (1 mM stock concentration in KPhos pH 7.4), 1.5 µL of 1 mM VH032 azide (stock in DMF), 1 uL of 200 mM DIPEA, 4.5 µL of H_2O, 1 µL of 200 mM sodium ascorbate and finally, 1 µL of 200 mM $CuBrMe_2S$ (stock in DMSO). The reaction was vortexed, spun down in a tabletop centrifuge and placed in a Thermomixer (Eppendorf) overnight, shaking at 800 rpm at room temperature.

The reaction was quenched with 250 µL of 0.5 M EDTA pH 8, made up to 500 µL with H_2O and first run through a Amicon 3K 0.5 mL column/tube as described above to chelate excess copper and remove it along with the unreacted small molecule. The final eluent collected after inversion was then purified by HPLC on an Agilent Polaris C18 column (150 x 4.6 mm), column heated to 50°C using a gradient of 3-30% or 3-50% CH_3CN (indicated in **SI** figures) over 20 minutes, flow rate of 1.5 mL/min, with 10 mM triethylammonium bicarbonate (TEAB) pH 8.5 as an ion-pairing buffer throughout.

Alternate ***fully aqueous*** *reaction conditions for copper click chemistry*

To a 0.5 mL Eppendorf DNA LoBind tube was added, listed in order of addition, 1 µL of the DNA (1 mM stock concentration in KPhos pH 7.4), 1.5 µL of 1 mM JQ1-alkyne or VH032 azide (stock in DMF), 0.4 µL of 100 mM sodium ascorbate, 1.6 µL of H_2O, and finally, 0.5 µL of 50 mM $CuSO_4$:THPTA (pre-complexed, each component at a concentration of 50 mM, in a ratio of 1:1). The reaction was vortexed, spun down in a tabletop centrifuge and placed in a Thermomixer (Eppendorf) overnight, shaking at 800 rpm at room temperature.

Assembly of OligoPROTAC

3'-(+)-JQ1 ssDNA OligoPROTAC strand and complementary 5'-VHL032 ssDNA OligoPROTAC strand were combined in an equimolar ratio at 50µM in buffer: 30 mM HEPES pH 7.5, 100 mM CH_3COOK. The solution was then heated to 95°C in a thermocycler and slowly cooled down to 25°C over 40 minutes. Duplex formation was verified by running a native PAGE gel.

Oligonucleotide MS characterisation

Oligonucleotide Mass Spectra were recorded on a Waters Xevo G2 QTOF ESI-UPLC-MS system. A gradient of MeOH in Et_3N and hexafluoroisopropanol (HFIP) was used (buffer A, 8.6 mM Et_3N, 200 mM HFIP in 5% MeOH/H2O (v/v); buffer B, 20% buffer A in MeOH). Data was then deconvoluted using MassLynx software v4.1 and recorded in **table 2**.

Nucleic acid sequences

All unmodified Oligonucleotides were purchased from IDT as desalted and lyophilised products – resuspended in 10 mM Tris pH 8. Amine-containing Oligonucleotides were purchased from IDT, HPLC-purified in lyophilised form and dissolved in 10 mM potassium phosphate buffer, pH 8.

Table 1: Sequences for OligoPROTAC ssDNA strands, toehold (highlighted in red) (+)-JQ1 ssDNA strand along with anti-toehold and scrambled anti-toehold complement used for all transfection experiments.

* denotes phosphorothioate linkages

\Azide\ denotes azide modification

\Alkyne\ denotes alkyne modification

S.No.	Name	Sequence (5'-3')
1	(+)-JQ1 ssDNA OligoPROTAC, [n=0]	T*C*G*T*G*G*G*T*A*G*G*T*C*C*G*C*A*C*T*A\Azide\
2	VH032 ssDNA OligoPROTAC, [n=0]	\Alkyne\T*A*G*T*G*C*G*G*A*C*C*T*A*C*C*C*A*C*G*A
3	(+)-JQ1 ssDNA OligoPROTAC, [n=1]	T*C*G*T*G*G*G*T*A*G*G*T*C*C*G*C*A*C*T*A*T\Azide\
4	VH032 ssDNA OligoPROTAC, [n=1]	\Alkyne\T*T*A*G*T*G*C*G*G*A*C*C*T*A*C*C*C*A*C*G*A
5	(+)-JQ1 ssDNA OligoPROTAC, [n=2]	T*C*G*T*G*G*G*T*A*G*G*T*C*C*G*C*A*C*T*A*T*T\Azide\
6	VH032 ssDNA OligoPROTAC, [n=2]	\Alkyne\T*T*T*A*G*T*G*C*G*G*A*C*C*T*A*C*C*C*A*C*G*A
7	(+)-JQ1 ssDNA OligoPROTAC, [n=3]	T*C*G*T*G*G*G*T*A*G*G*T*C*C*G*C*A*C*T*A*T*T*T\Azide\
8	VH032 ssDNA OligoPROTAC, [n=3]	\Alkyne\T*T*T*T*A*G*T*G*C*G*G*A*C*C*T*A*C*C*C*A*C*G*A
9	(+)-JQ1 ssDNA OligoPROTAC, [n=5]	T*C*G*T*G*G*G*T*A*G*G*T*C*C*G*C*A*C*T*A*T*T*T*T*T\Azide\
10	VH032 ssDNA OligoPROTAC, [n=5]	\Alkyne\T*T*T*T*T*T*A*G*T*G*C*G*G*A*C*C*T*A*C*C*C*A*C*G*A
11	(+)-JQ1 ssDNA OligoPROTAC, [n=10]	T*C*G*T*G*G*G*T*A*G*G*T*C*C*G*C*A*C*T*A*T*T*T*T*T*T*T*T*T*T\Azide\
12	VH032 ssDNA OligoPROTAC, [n=10]	\Alkyne\T*T*T*T*T*T*T*T*T*T*T*A*G*T*G*C*G*G*A*C*C*T*A*C*C*C*A*C*G*A
13	(8-base pair) Toehold-(+)-JQ1 ssDNA, [n=3]	T*C*A*A*C*A*T*C*T*C*G*T*G*G*G*T*A*G*G*T*C*C*G*C*A*C*T*A*T*T*T\Azide\
14	(8-base pair) Anti-toehold complement	A*A*A*T*A*G*T*G*C*G*G*A*C*C*T*A*C*C*C*A*C*G*A*G*A*T*G*T*T*G*A*
15	(8-base pair) Scrambled anti-toehold complement	A*A*C*A*A*T*C*A*C*T*T*C*T*A*A*C*A*A*T*T*C*C*T*T*T*T*C*C*

16	(7-base pair) Toehold ssDNA, [n=3]	T*C*A*A*C*A*T*T*C*G*T*G*G*G*T*A*G*G*T*C*C*G*C*A*C*T*A*T*T*T
17	(9-base pair) Toehold ssDNA, [n=3]	T*C*A*A*C*A*T*C*A*T*C*G*T*G*G*G*T*A*G*G*T*C*C*G*C*A*C*T*A*T*T*T
18	(10-base pair) Toehold ssDNA, [n=3]	T*C*A*A*C*A*T*C*A*G*T*C*G*T*G*G*G*T*A*G*G*T*C*C*G*C*A*C*T*A*T*T*T
19	(7-base pair) Anti-toehold complement	A*A*A*T*A*G*T*G*C*G*G*A*C*C*T*A*C*C*C*A*C*G*A*A*T*G*T*T*G*A*
20	(9-base pair) Anti-toehold complement	A*A*A*T*A*G*T*G*C*G*G*A*C*C*T*A*C*C*C*A*C*G*A*T*G*A*T*G*T*T*G*A
21	(10-base pair) Anti-toehold complement	A*A*A*T*A*G*T*G*C*G*G*A*C*C*T*A*C*C*C*A*C*G*A*C*T*G*A*T*G*T*T*G*A

Table 2: Molecular weights for Oligonucleotide conjugates prepared

S.No.	Name	Expected mass	Mass after deconvolution
1	(+)-JQ1 ssDNA OligoPROTAC, [n=0]	7238.5	**7239.5**
2	VH032 ssDNA OligoPROTAC, [n=0]	7108.7	**7109.5**
3	(+)-JQ1 ssDNA OligoPROTAC, [n=1]	7563.0	**7561.0**
4	VH032 ssDNA OligoPROTAC, [n=1]	7429.7	**7429.0**
5	(+)-JQ1 ssDNA OligoPROTAC, [n=2]	7888.5	**7882.5**
6	VH032 ssDNA OligoPROTAC, [n=2]	7751.2	**7751.0**
7	(+)-JQ1 ssDNA OligoPROTAC, [n=3]	8206.5	**8201.5**
8	VH032 ssDNA OligoPROTAC, [n=3]	8070.2	**8068.0**
9	(+)-JQ1 ssDNA OligoPROTAC, [n=5]	8841.0	**8842.5**
10	VH032 ssDNA OligoPROTAC, [n=5]	8710.0	**8710.5**
11	(+)-JQ1 ssDNA OligoPROTAC, [n=10]	10442.0	**10445.0**
12	VH032 ssDNA OligoPROTAC, [n=10]	10311.2	**10312.5**
13	antisymmetric OligoPROTAC (+)-JQ1, [n=3]	8171.0	**8170.5**
14	Toehold-(+)-JQ1 ssDNA, [n=3]	10747.0	**10745.5**

Biological assays

Cell culture

HeLa pLuc/705 and HEK293T cells were both cultured in Dulbecco's Modified Eagle Medium with 1X GlutaMAX (Gibco) supplemented with 10% (v/v) FBS (Gibco) and) at 37 °C in a humidified incubator with 5% CO_2.

Transfection

For transfection with lipofectamine 2000, 2.5 × 10^5 HEK239T cells were plated in 1 mL of culture media in 24-well plates 16 hours before transfection to reach 70-80% cell confluency. Prior to transfection, the culture media was replaced with OptiMEM, 100 µL for the 96-well plates and 1 mL for the 24-well plates.

2 µL of Lipofectamine 2000 (Invitrogen) was added to 48 µL OptiMEM (Gibco) and incubated at room temperature for 5 minutes before mixing with 0.5 nmol of Oligonucleotide dissolved in 50 µL of OptiMEM. The resulting mixture was incubated at room temperature for 20 minutes allowing complexation to occur. The complexes were then added to the cells at the required concentrations (with total volume of 1 mL per well). The cells were then incubated at 37 °C in a

5% CO_2 incubator. After 6 hours the media was replaced with 1 mL of culture media and the cells were returned to the incubator for another 18 hours.

Western blotting

Salt-soluble proteins were extracted from 1×10^6 cells by incubating cells in a high-salt lysis buffer (20 mM Tris-HCl pH 8.0, 300 mM KCl, 5 mM EDTA, 20% glycerol, 0.5% IGEPAL CA-630, protease inhibitor cocktail). Protein extracts were then run on a NuPAGE 4-12% BisTris gels (Life Technologies) at 180V for 1 hour and blotted onto a polyvinylidene fluoride membrane (Immobilon) at 100V for 1 hour using a Tris-glycine blotting buffer3. The blots were then probed with 1:10,000 dilution of primary antibody for BRD4 (CST #13440, E2A7X) in 5% milk/TBS-tween at 4°C overnight. BRD4 blots were then probed with secondary antibody (ab216773, IRDye 800CW) for 2 hours (Figures **1b**, **4b**, SI Figures **14**, **15**, **16**, **17**, **23**) or secondary antibody (LiChor IRDye 800 CW) for 2 hours **(**Figures **3a**,**b**,**c**, **4c**, SI Figures **18**, **19**, **24)** at room temperature, followed by imaging on the ChemiDoc MP system.

Molecular Modelling

The structure of the PROTAC, MZ1 in complex with the second bromodomain of BRD4 ($BRD44^{BD2}$) and Von Hippel-Lindau disease tumor suppressor:ElonginC:ElonginB (PDB ID: 5T35[3]) was downloaded from the protein databank (www.rcsb.org). The structure was cleaned to remove transcription elongation factor polypeptide 1 (chain C) and 2 (chain B). The PEG linker from MZ1 was removed such that MZ1 was split into VH032 and (+)-JQ1. The warhead groups of VH032 and (+)-JQ1 were restrained so that the original conformation in the crystal structure was retained. The linkers were extended from the point of cleavage that attached VH032 to the 5'-end of one DNA Oligonucleotide and (+)-JQ1 to the 3'-end to the complementary DNA Oligonucleotide. A further 3 unpaired thymidines were added on each strand, [n=3], followed by an extension of a 20 bp duplex DNA containing the sequence AGCACCCATCCAGGCGTGAT on the 5'-3' strand and its complementary sequence on the 3'-5' strand. The final structure was minimized *in situ* for a 1000 steps conjugate gradient, to relieve any steric clashes between the side chains and the extended OligoPROTAC, [n=3]. All structural manipulation and construction of the molecular model was carried out in ICM-Pro suite software (www.molsoft.com).

Supplementary Figures 1-28

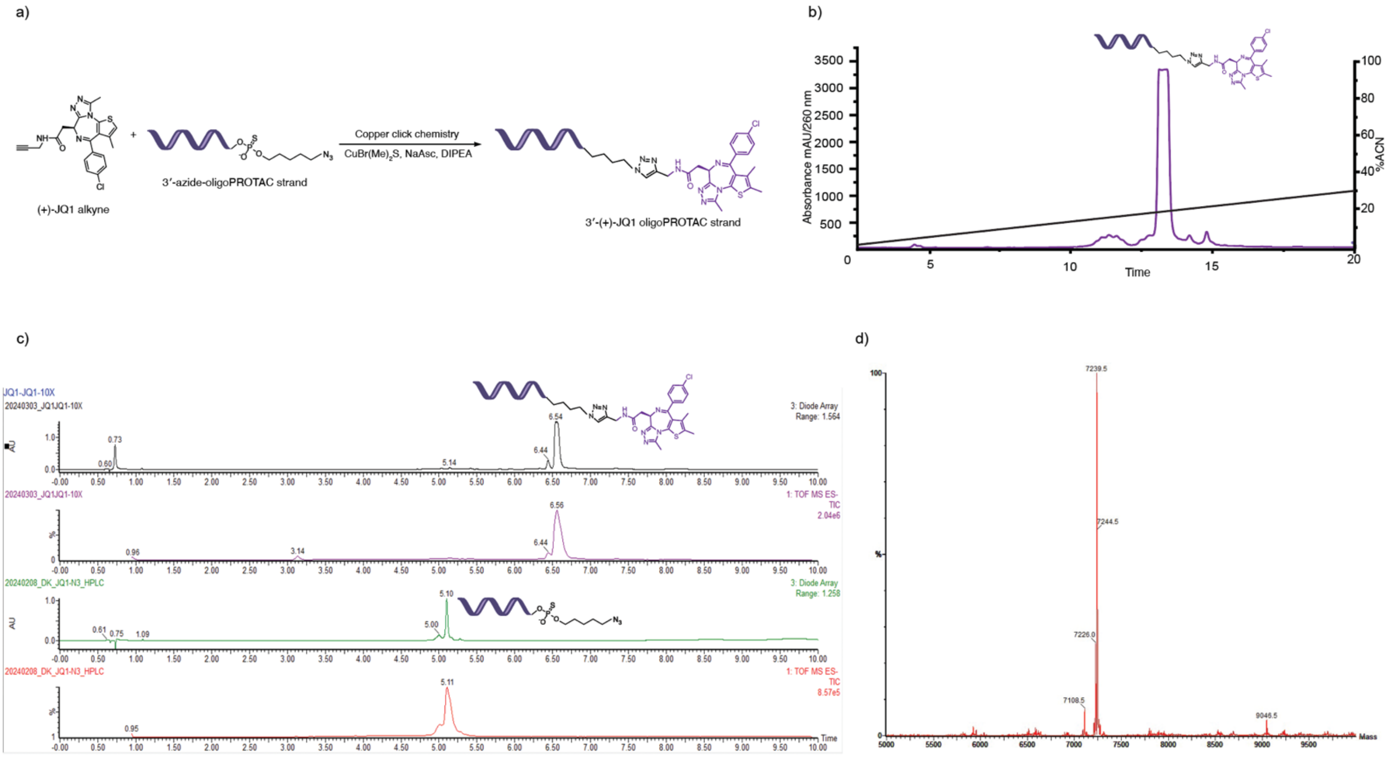

Supplementary Figure 1. Reaction and characterisation for (+)-JQ1 ssDNA OligoPROTAC, [n=0]. **a**) Reaction scheme for copper click conjugation of (+)-JQ1-alkyne with azide-OligoPROTAC, [n=0]. **b**) HPLC purification for (+)-JQ1 ssDNA OligoPROTAC, [n=0]. **c**) LC-MS characterisation for (+)-JQ1 ssDNA OligoPROTAC, [n=0]. **d**) Mass spectrum for HPLC-purified (+)-JQ1 ssDNA OligoPROTAC, [n=0].

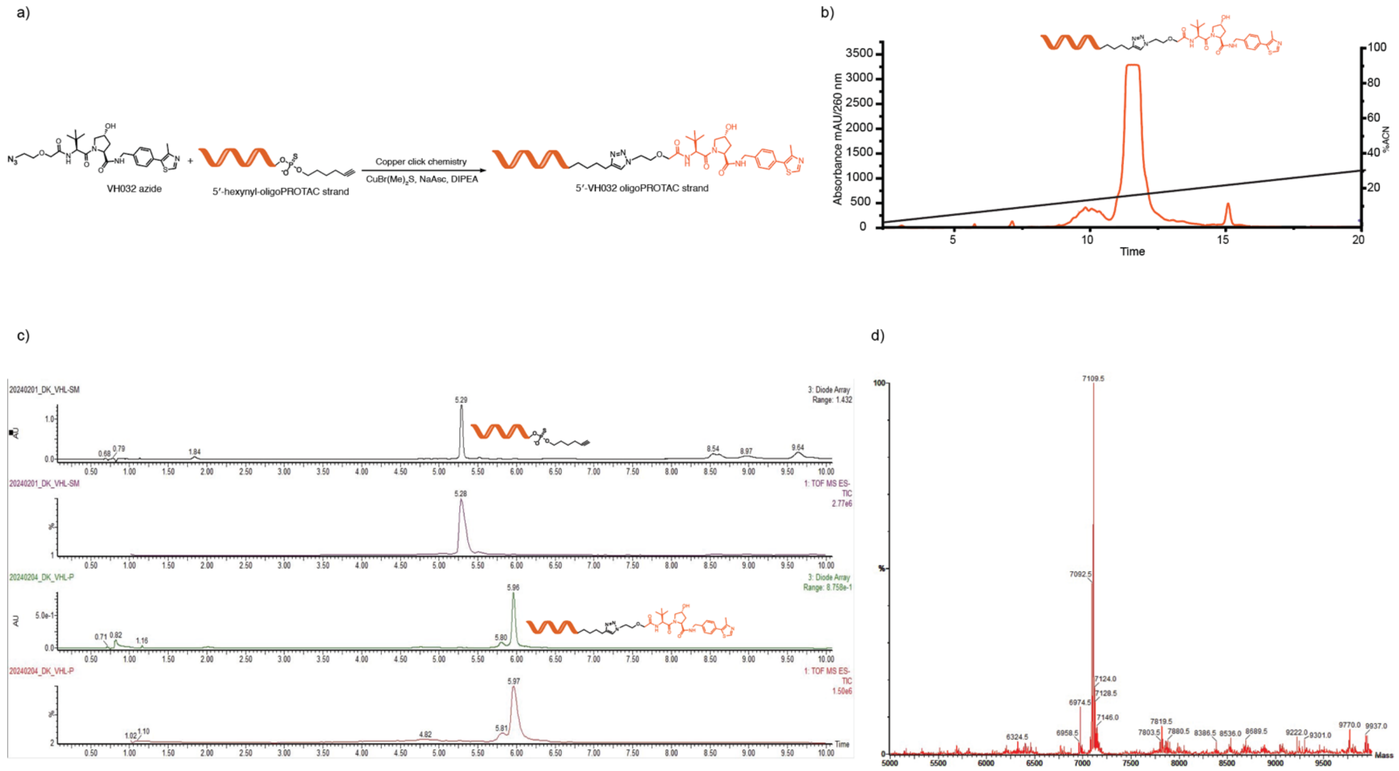

Supplementary Figure 2. Reaction and characterisation for VH032 ssDNA OligoPROTAC, [n=0]. **a**) Reaction scheme for copper click conjugation of VH032 azide with alkyne-OligoPROTAC, [n=0]. **b**) HPLC purification for VH032 ssDNA OligoPROTAC, [n=0]. **c**) LC-MS characterisation for VH032 ssDNA OligoPROTAC, [n=0]. **d**) Mass spectrum for HPLC-purified VH032 ssDNA OligoPROTAC, [n=0].

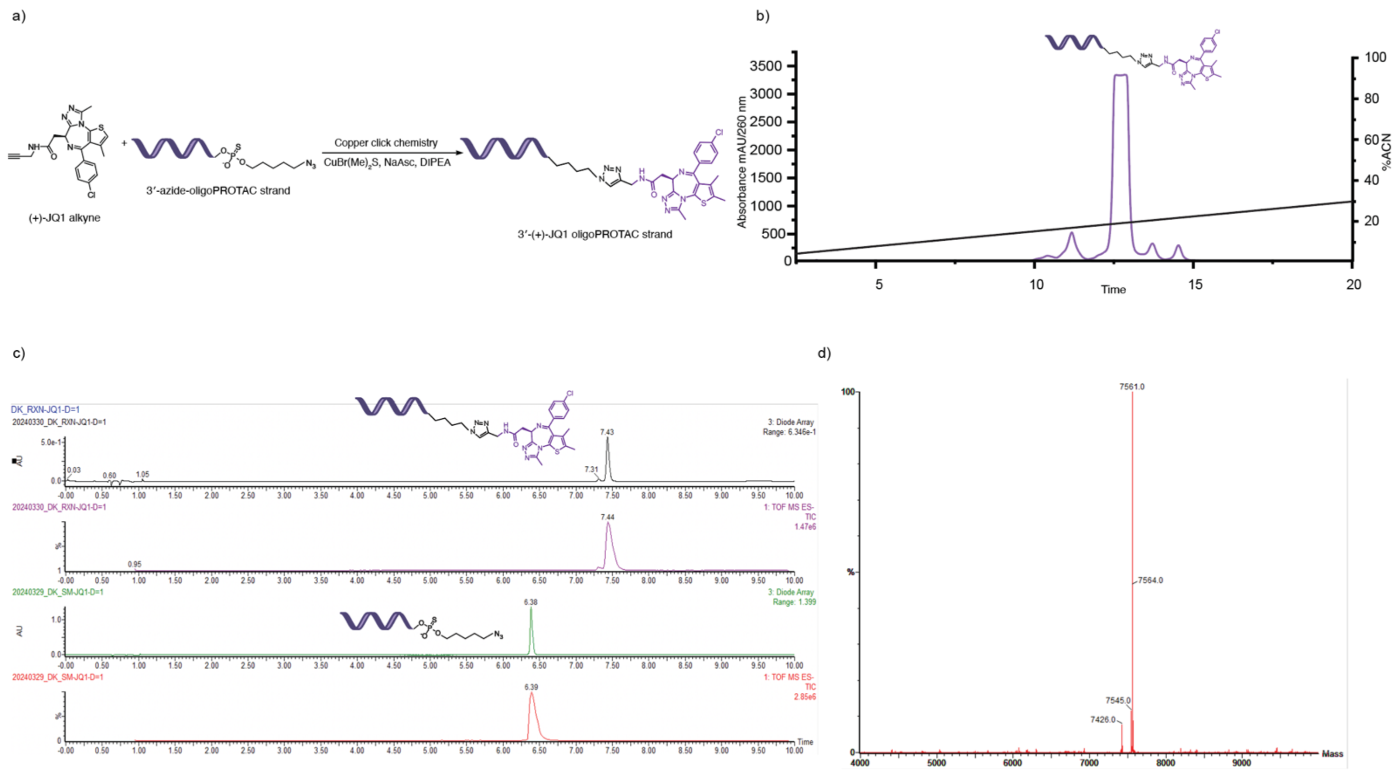

Supplementary Figure 3. Reaction and characterisation for (+)-JQ1 ssDNA OligoPROTAC, [n=1]. **a**) Reaction scheme for copper click conjugation of (+)-JQ1-alkyne with azide-OligoPROTAC, [n=1]. **b**) HPLC purification for (+)-JQ1 ssDNA OligoPROTAC, [n=1]. **c**) LC-MS characterisation for (+)-JQ1 ssDNA OligoPROTAC, [n=1]. **d**) Mass spectrum for HPLC-purified (+)-JQ1 ssDNA OligoPROTAC, [n=1].

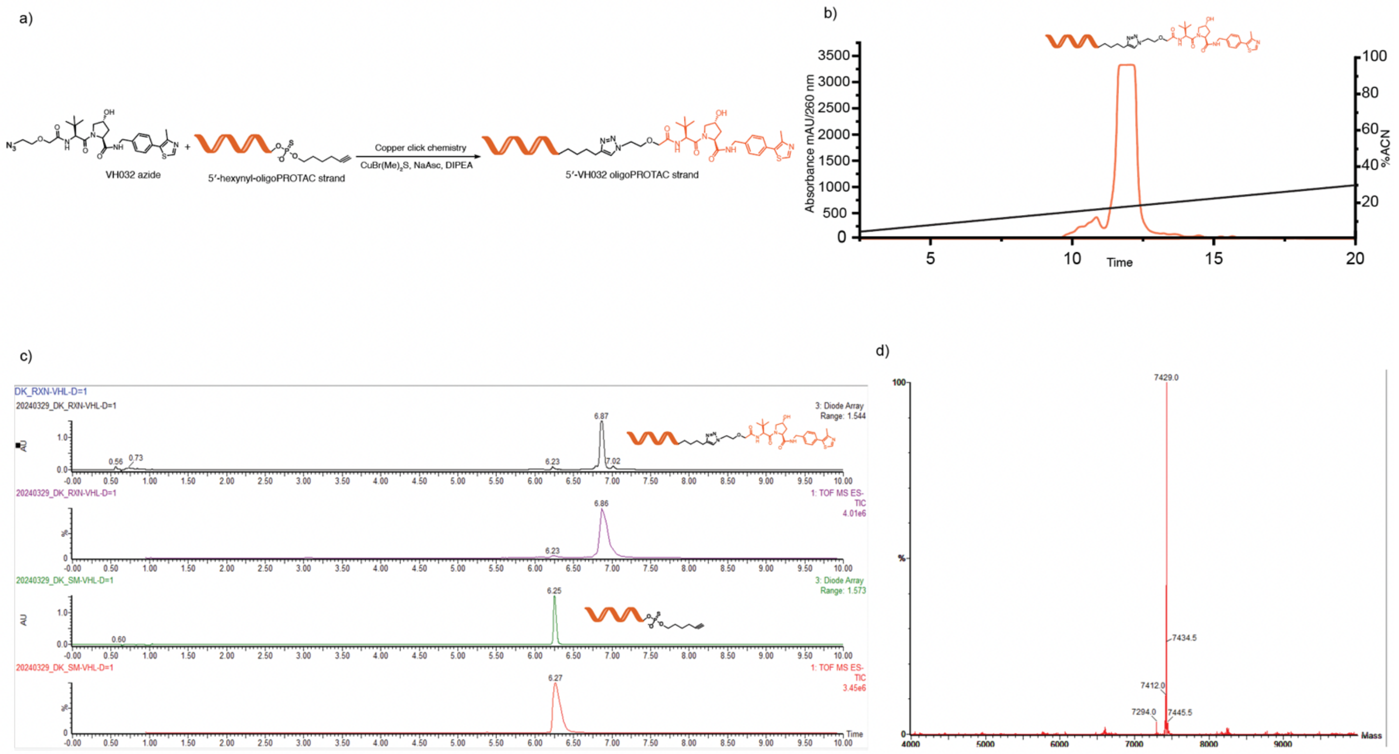

Supplementary Figure 4. Reaction and characterisation for VH032 ssDNA OligoPROTAC, [n=1]. **a**) Reaction scheme for copper click conjugation of VH032 azide with alkyne-OligoPROTAC, [n=1]. **b**) HPLC purification for VH032 ssDNA OligoPROTAC, [n=1]. **c**) LC-MS characterisation for VH032 ssDNA OligoPROTAC, [n=1]. **d**) Mass spectrum for HPLC-purified VH032 ssDNA OligoPROTAC, [n=1].

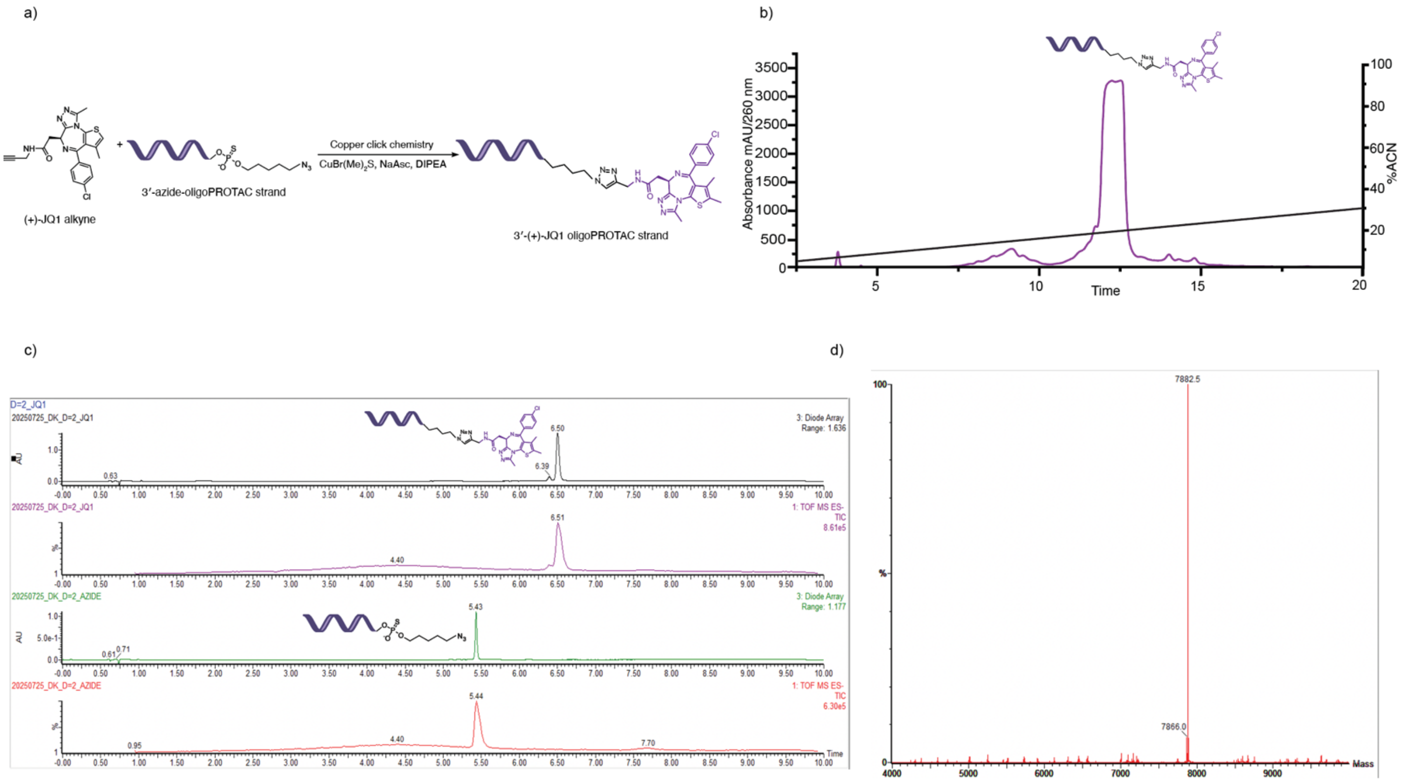

Supplementary Figure 5. Reaction and characterisation for (+)-JQ1 ssDNA OligoPROTAC, [n=2]. **a**) Reaction scheme for copper click conjugation of (+)-JQ1-alkyne with azide-OligoPROTAC, [n=2]. **b**) HPLC purification for (+)-JQ1 ssDNA OligoPROTAC, [n=2]. **c**) LC-MS characterisation for (+)-JQ1 ssDNA OligoPROTAC, [n=2]. **d**) Mass spectrum for HPLC-purified (+)-JQ1 ssDNA OligoPROTAC, [n=2].

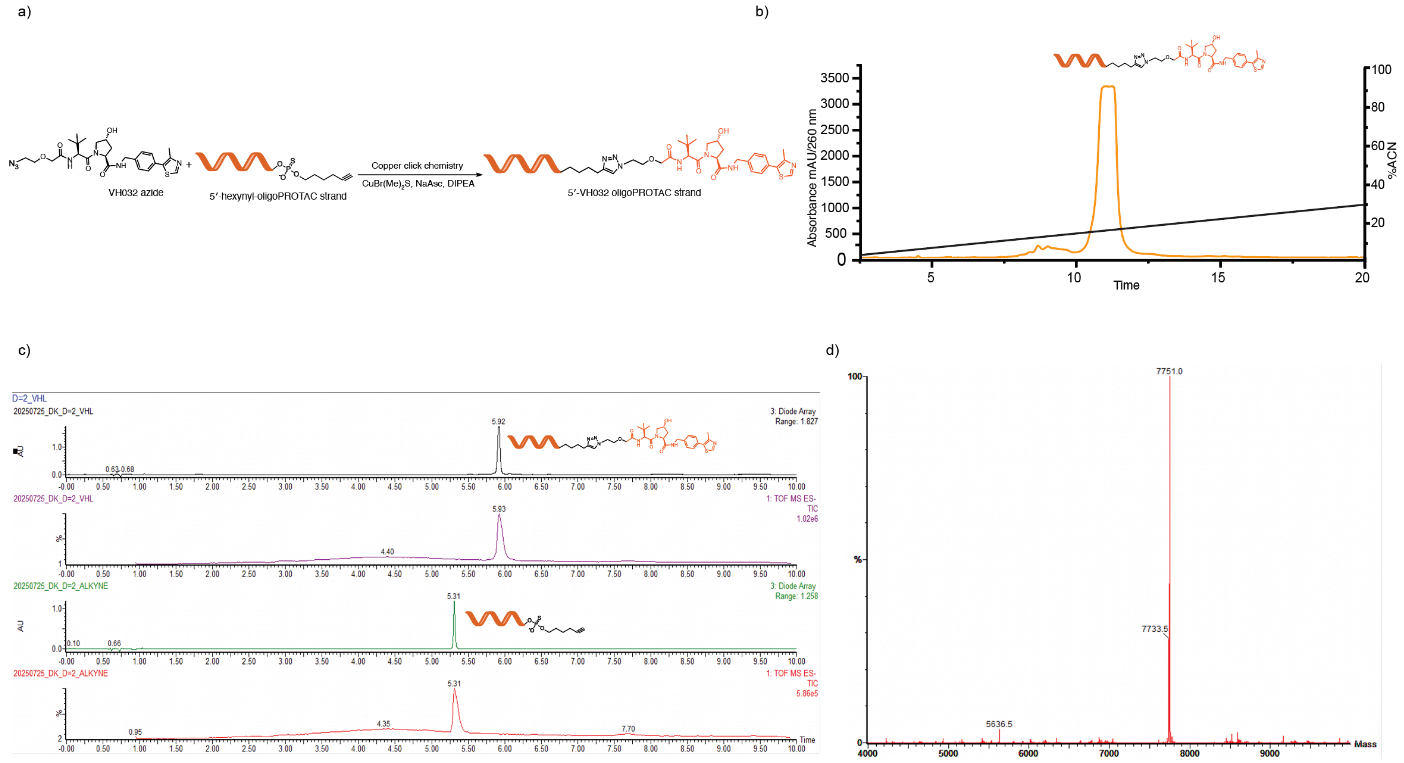

Supplementary Figure 6. Reaction and characterisation for VH032 ssDNA OligoPROTAC, [n=2]. **a**) Reaction scheme for copper click conjugation of VH032 azide with alkyne-OligoPROTAC, [n=2]. **b**) HPLC purification for VH032 ssDNA OligoPROTAC, [n=2]. **c**) LC-MS characterisation for VH032 ssDNA OligoPROTAC, [n=2]. **d**) Mass spectrum for HPLC-purified VH032 ssDNA OligoPROTAC, [n=2].

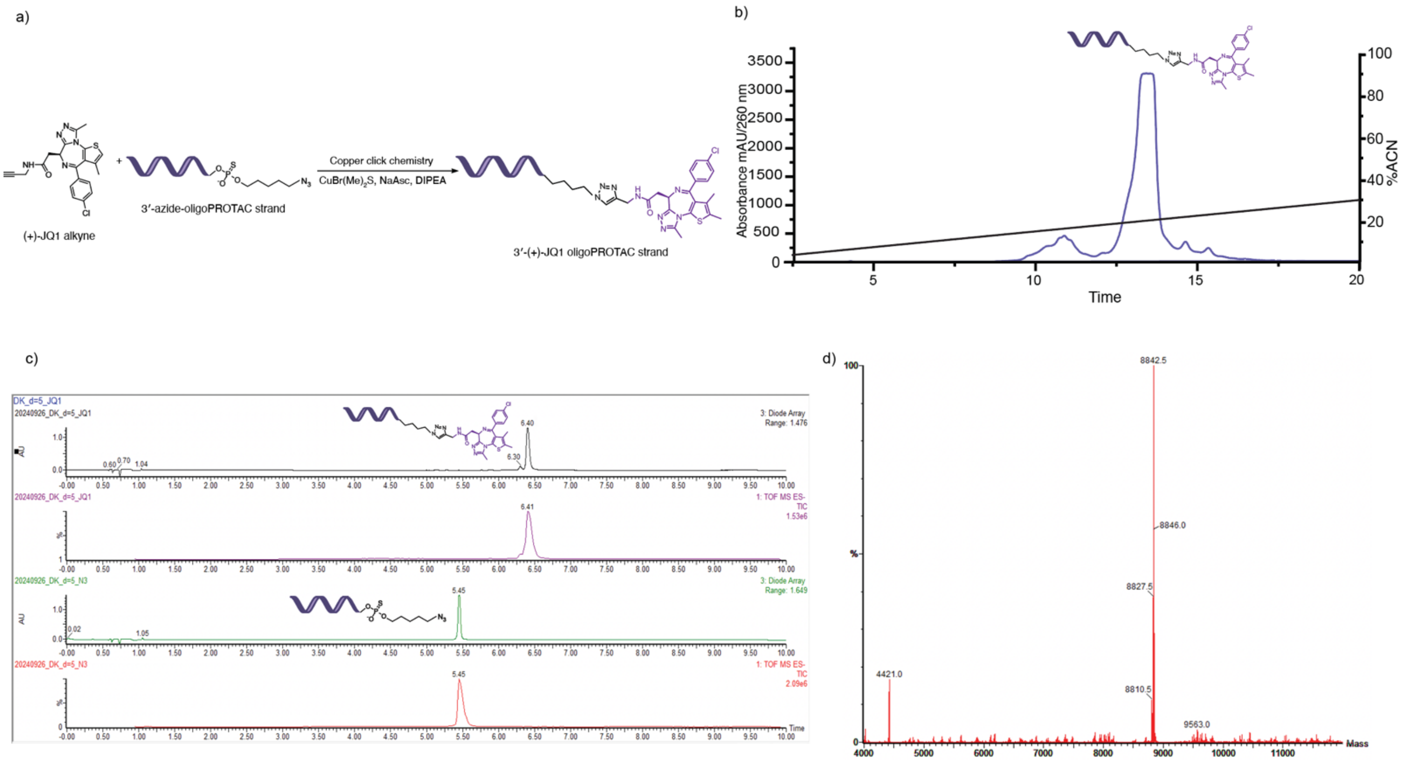

Supplementary Figure 7. Reaction and characterisation for (+)-JQ1 ssDNA OligoPROTAC, [n=3]. **a**) Reaction scheme for copper click conjugation of (+)-JQ1-alkyne with azide-OligoPROTAC, [n=3]. **b**) HPLC purification for (+)-JQ1 ssDNA OligoPROTAC, [n=3]. **c**) LC-MS characterisation for (+)-JQ1 ssDNA OligoPROTAC, [n=3]. **d**) Mass spectrum for HPLC-purified (+)-JQ1 ssDNA OligoPROTAC, [n=3].

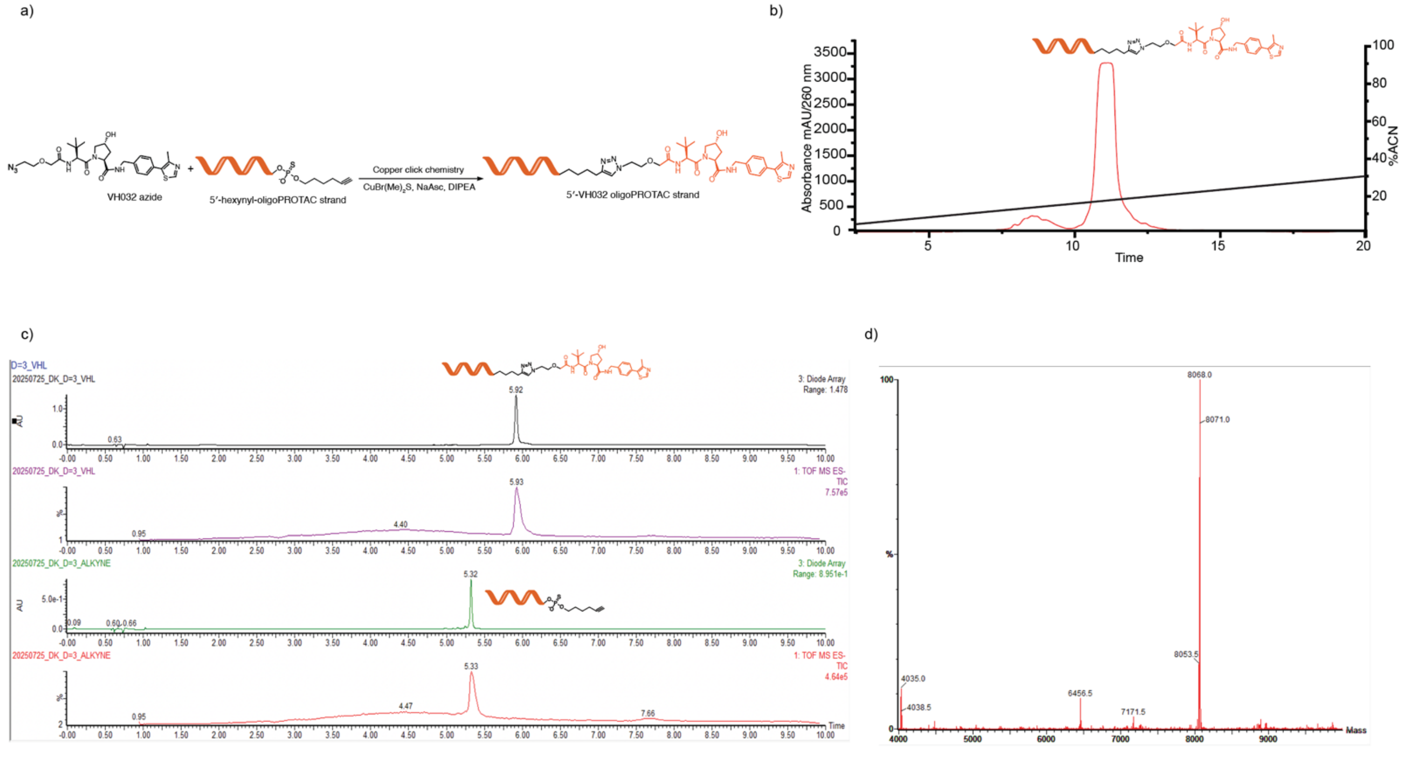

Supplementary Figure 8. Reaction and characterisation for VH032 ssDNA OligoPROTAC, [n=3]. **a**) Reaction scheme for copper click conjugation of VH032 azide with alkyne-OligoPROTAC, [n=3]. **b**) HPLC purification for VH032 ssDNA OligoPROTAC, [n=3]. **c**) LC-MS characterisation for VH032 ssDNA OligoPROTAC, [n=3]. **d**) Mass spectrum for HPLC-purified VH032 ssDNA OligoPROTAC, [n=3].

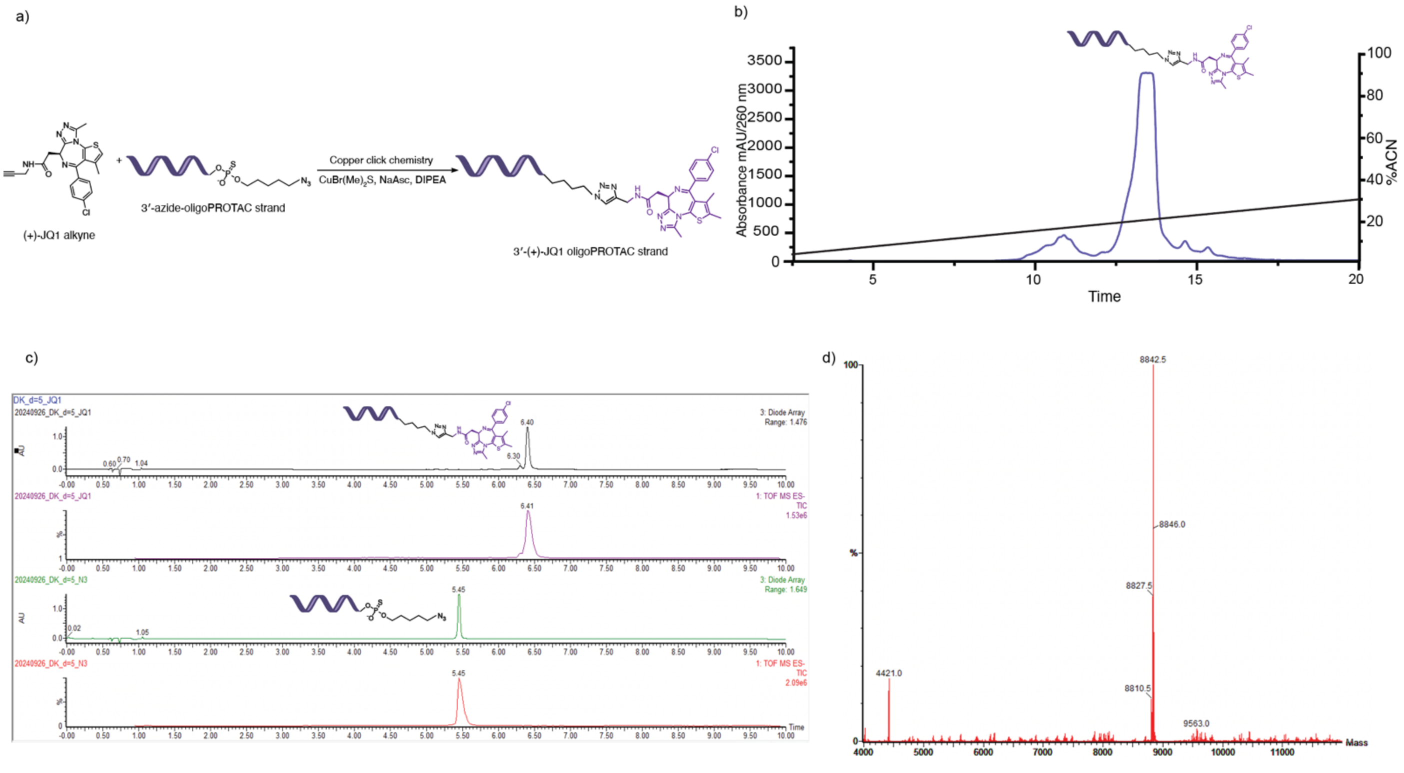

Supplementary Figure 9. Reaction and characterisation for (+)-JQ1 ssDNA OligoPROTAC, [n=5]. **a**) Reaction scheme for copper click conjugation of (+)-JQ1-alkyne with azide-OligoPROTAC, [n=5]. **b**) HPLC purification for (+)-JQ1 ssDNA OligoPROTAC, [n=5]. **c**) LC-MS characterisation for (+)-JQ1 ssDNA OligoPROTAC, [n=5]. **d**) Mass spectrum for HPLC-purified (+)-JQ1 ssDNA OligoPROTAC, [n=5].

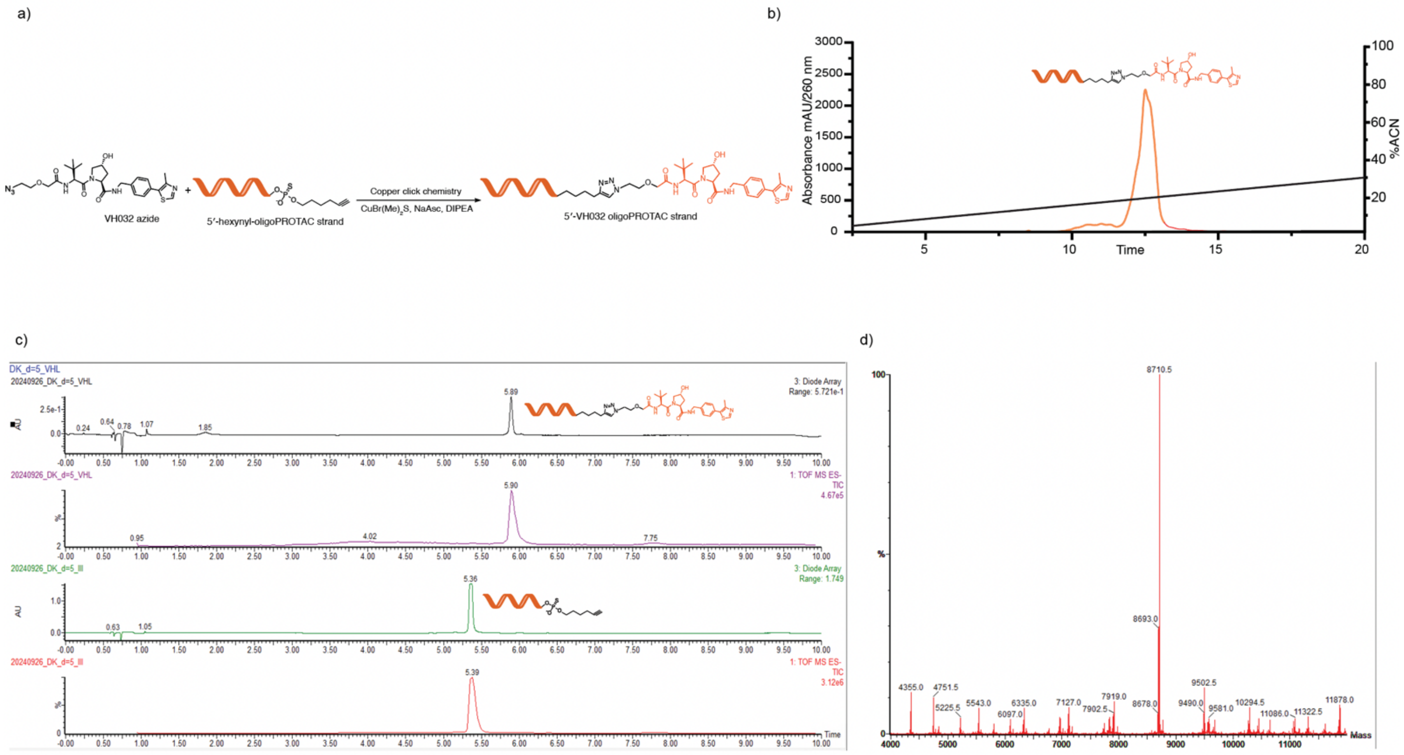

Supplementary Figure 10. Reaction and characterisation for VH032 ssDNA OligoPROTAC, [n=5]. **a**) Reaction scheme for copper click conjugation of VH032 azide with alkyne-OligoPROTAC, [n=5]. **b**) HPLC purification for VH032 ssDNA OligoPROTAC, [n=5]. **c**) LC-MS characterisation for VH032 ssDNA OligoPROTAC, [n=5]. **d**) Mass spectrum for HPLC-purified VH032 ssDNA OligoPROTAC, [n=5].

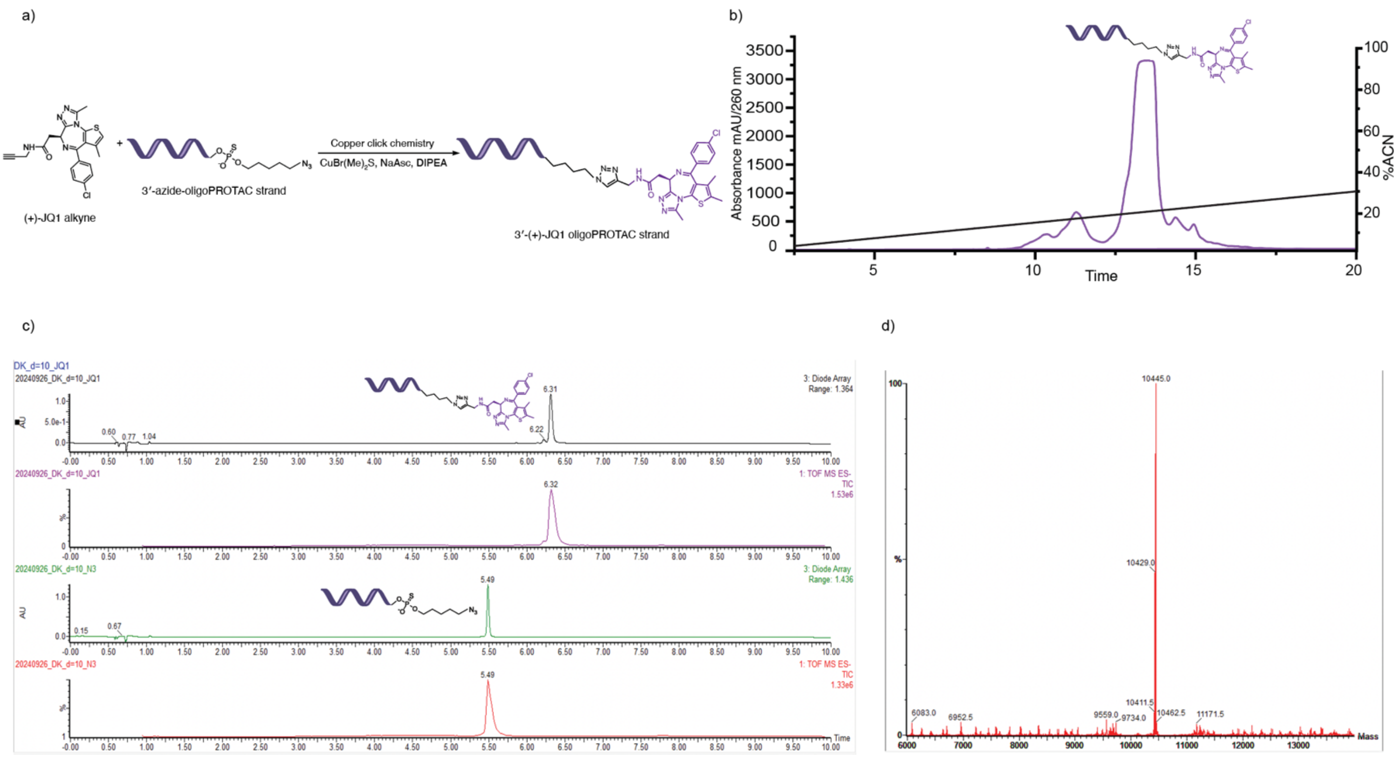

Supplementary Figure 11. Reaction and characterisation for (+)-JQ1 ssDNA OligoPROTAC, [n=10]. **a**) Reaction scheme for copper click conjugation of (+)-JQ1-alkyne with azide-OligoPROTAC, [n=10]. **b**) HPLC purification for (+)-JQ1 ssDNA OligoPROTAC, [n=10]. **c**) LC-MS characterisation for (+)-JQ1 ssDNA OligoPROTAC, [n=10]. **d**) Mass spectrum for HPLC-purified (+)-JQ1 ssDNA OligoPROTAC, [n=10].

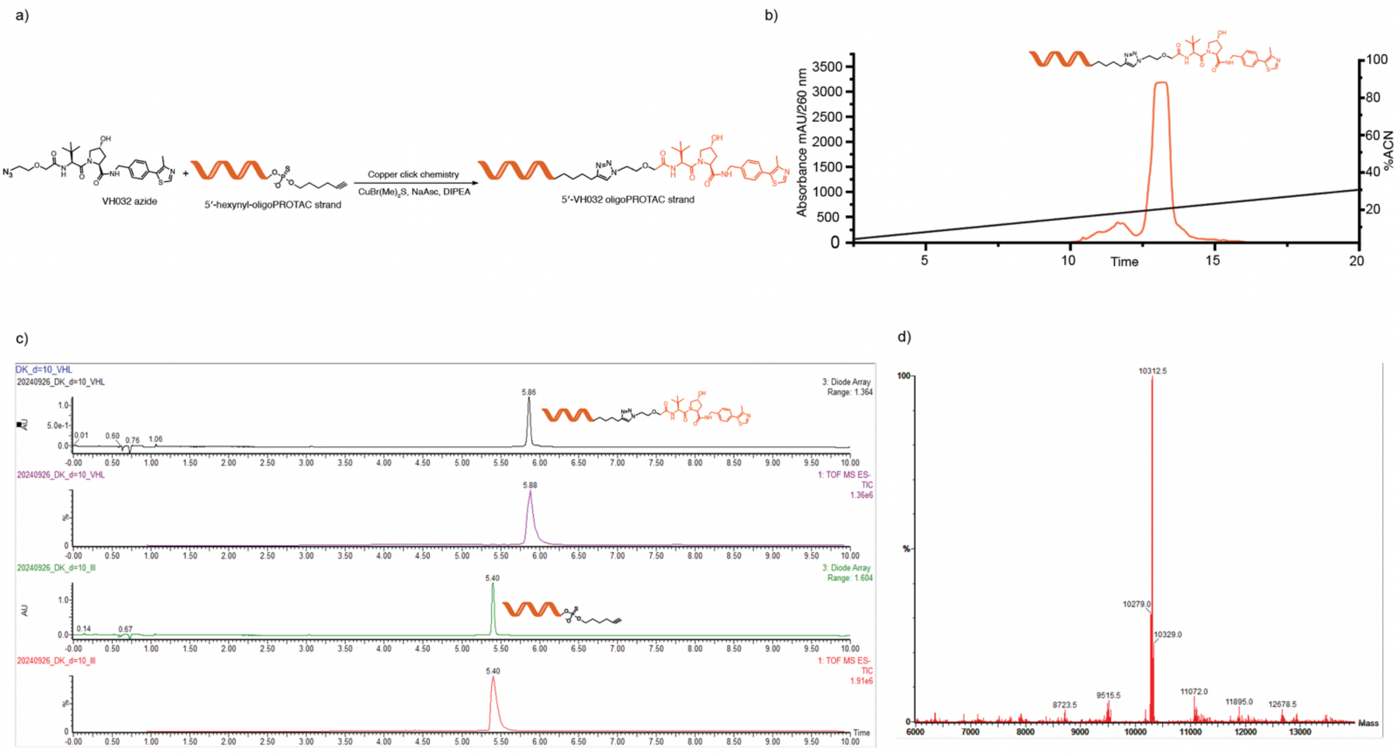

Supplementary Figure 12. Reaction and characterisation for VH032 ssDNA OligoPROTAC, [n=10]. **a**) Reaction scheme for copper click conjugation of VH032 azide with alkyne-OligoPROTAC, [n=10]. **b**) HPLC purification for VH032 ssDNA OligoPROTAC, [n=10]. **c**) LC-MS characterisation for VH032 ssDNA OligoPROTAC, [n=10]. **d**) Mass spectrum for HPLC-purified VH032 ssDNA OligoPROTAC, [n=10].

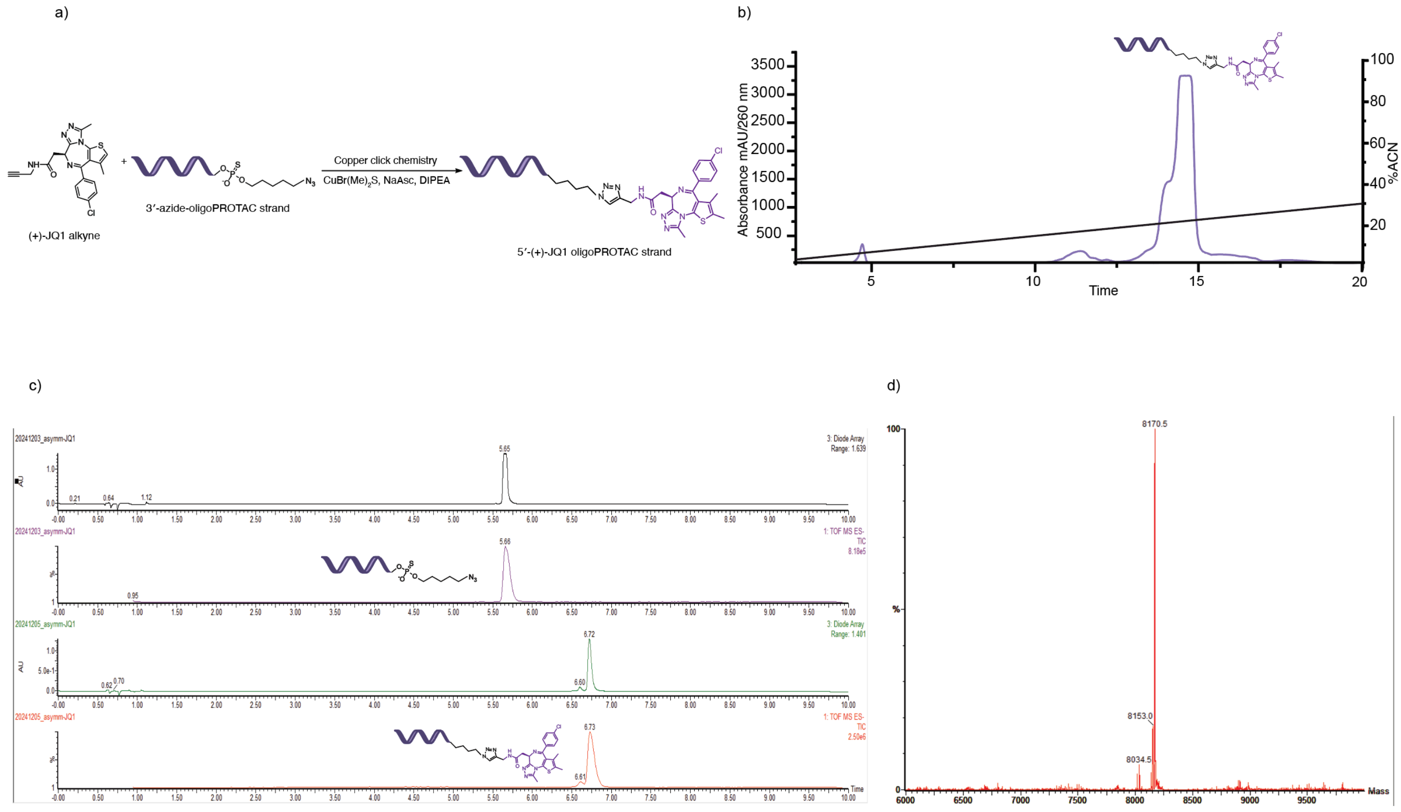

Supplementary Figure 13. Reaction and characterisation for (+)-JQ1 asymmetric ssDNA OligoPROTAC, [n=3]. **a**) Reaction scheme for copper click conjugation of (+)-JQ1-alkyne with 5'-azide-OligoPROTAC, [n=10]. **b**) HPLC purification for (+)-JQ1 asymmetric ssDNA OligoPROTAC, [n=3]. **c**) LC-MS characterisation for (+)-JQ1 asymmetric ssDNA OligoPROTAC, [n=3]. **d**) Mass spectrum for HPLC-purified (+)-JQ1 asymmetric ssDNA OligoPROTAC, [n=3].

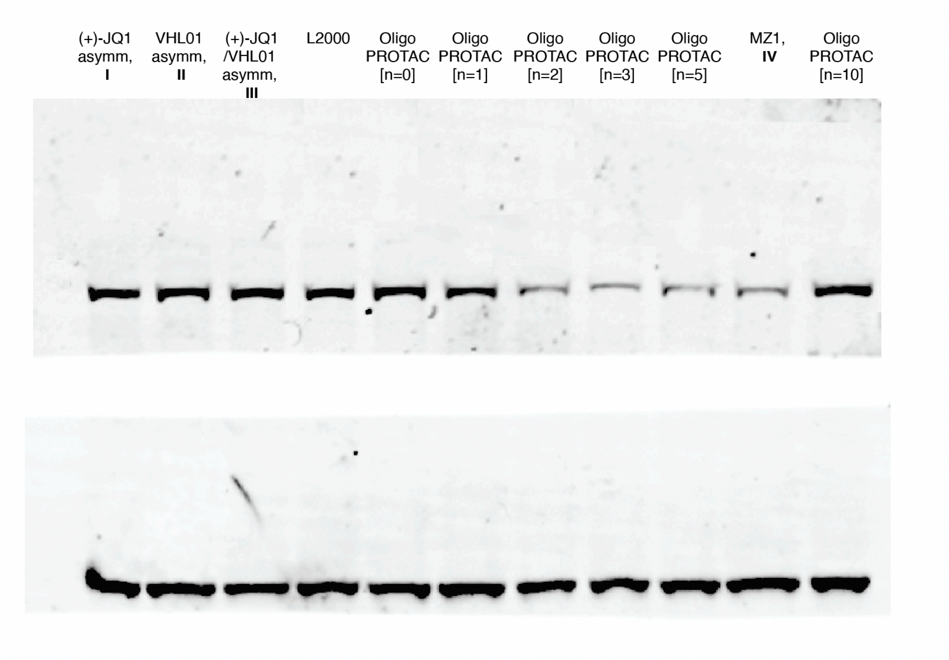

Supplementary Figure 14. Uncropped western blot of BRD4 levels in HEK293T upon treatment with OligoPROTAC of varying linker lengths and partial PROTAC control constructs.

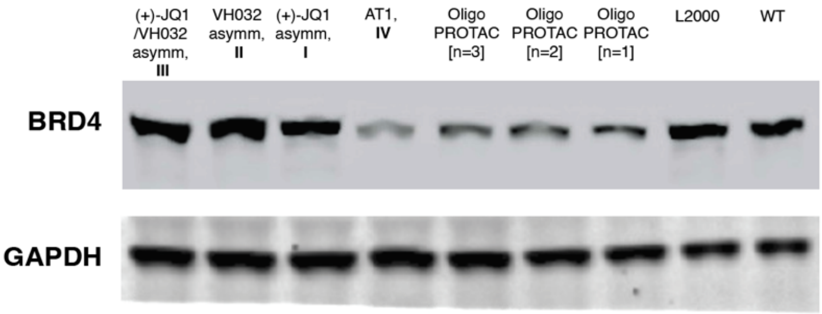

Supplementary Figure 15. Uncropped western blot of BRD4 levels in HeLa upon treatment with OligoPROTAC of varying linker lengths and partial PROTAC control constructs.

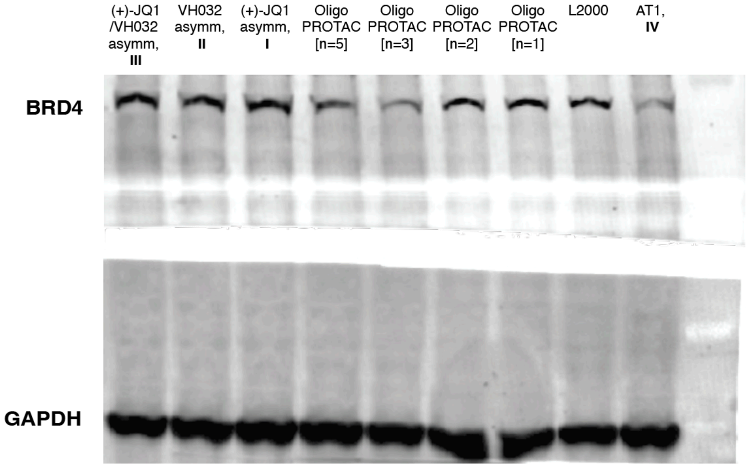

Supplementary Figure 16. Uncropped western blot of BRD4 levels in A549 upon treatment with OligoPROTAC of varying linker lengths and partial PROTAC control constructs.

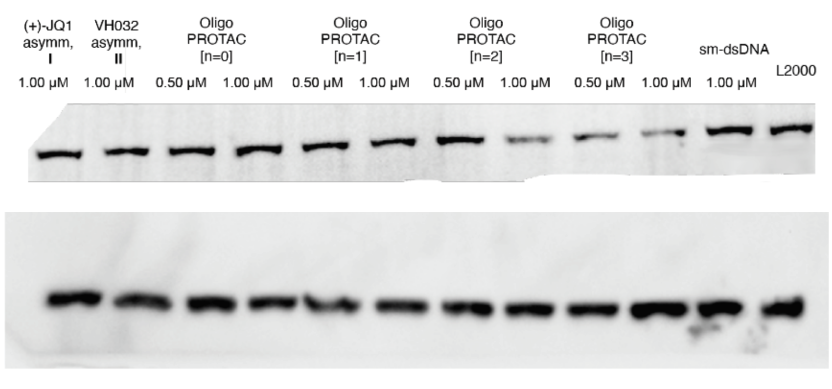

Supplementary Figure 17. Uncropped western blot of BRD4 levels upon lipofectamine 2000 transfection in HEK293T with OligoPROTAC of varying linker lengths, [n=1, 2, 3] and partial PROTAC control constructs

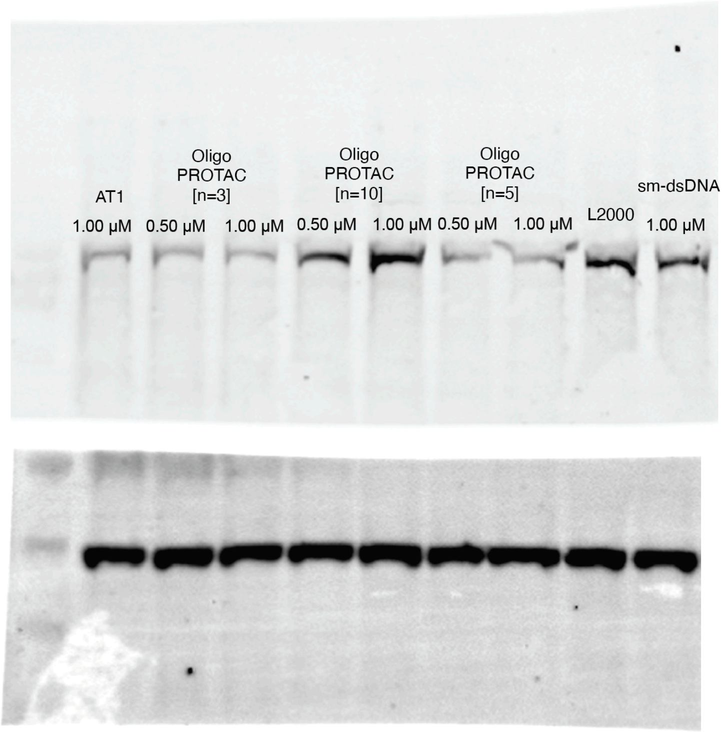

Supplementary Figure 18. Uncropped western blot of BRD4 levels upon lipofectamine 2000 transfection in HEK293T with OligoPROTAC of varying linker lengths, [n=3, 5, 10] and control constructs

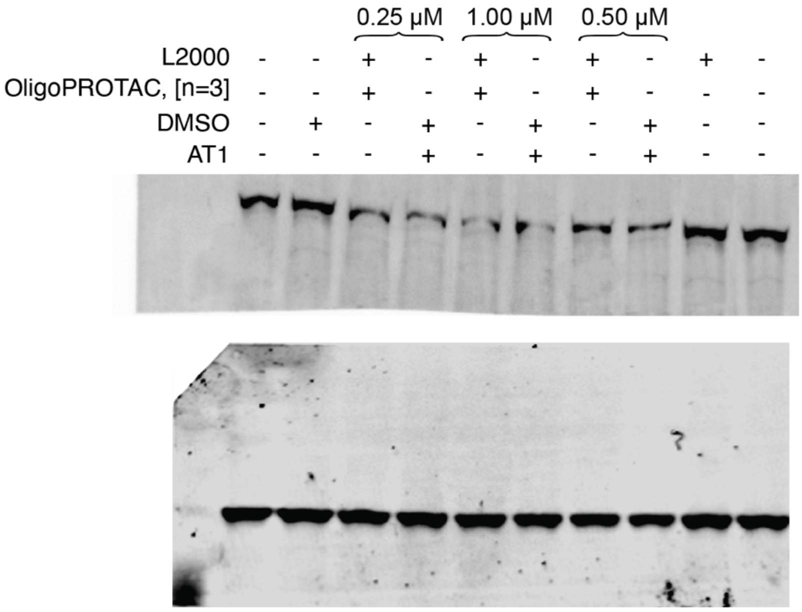

Supplementary Figure 19. Uncropped western blot of BRD4 levels upon treatment with n=3 OligoPROTAC and small molecule PROTAC, AT1 at concentrations indicated over upon Lipofectamine 2000 transfection/DMSO treatment in HEK293T cells at 12 hours. Normalised to GAPDH levels.

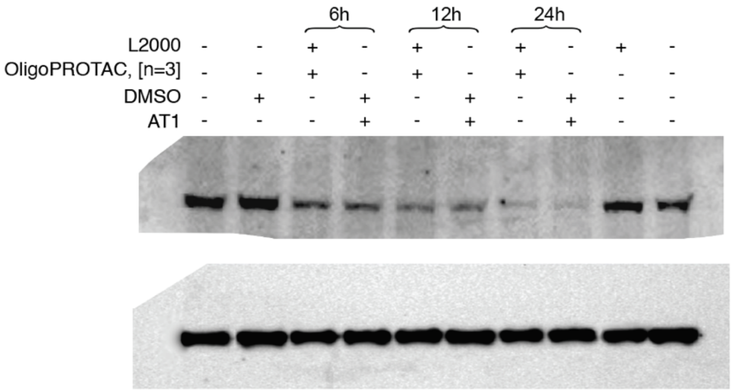

Supplementary Figure 20. Uncropped western blot for BRD4 levels upon treatment with n=3 OligoPROTAC and small molecule PROTAC, AT1 at 1.00 µM over 6 hours, 12 hours, and 24 hours upon Lipofectamine 2000 transfection/DMSO treatment in HEK293T cells. Normalised to GAPDH levels.

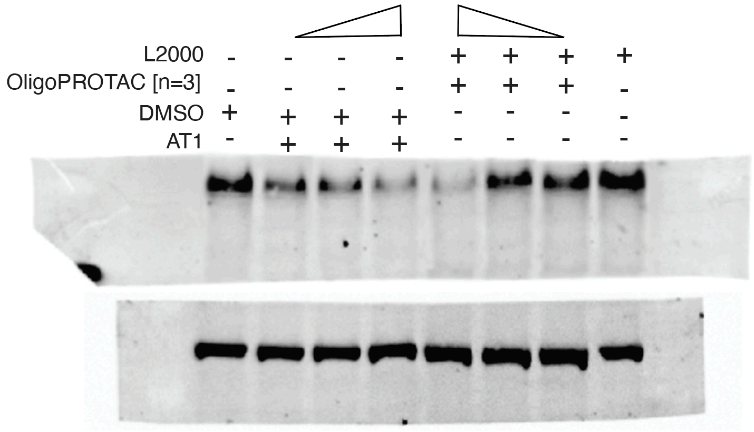

Supplementary Figure 21. Uncropped western blot Western blot for MYC levels upon treatment with n=3 OligoPROTAC and small molecule PROTAC, AT1 at concentrations (0.25, 0.50, 1.00 µM) over upon Lipofectamine 2000 transfection/DMSO treatment in HEK293T cells at 12 hours. Normalised to GAPDH levels.

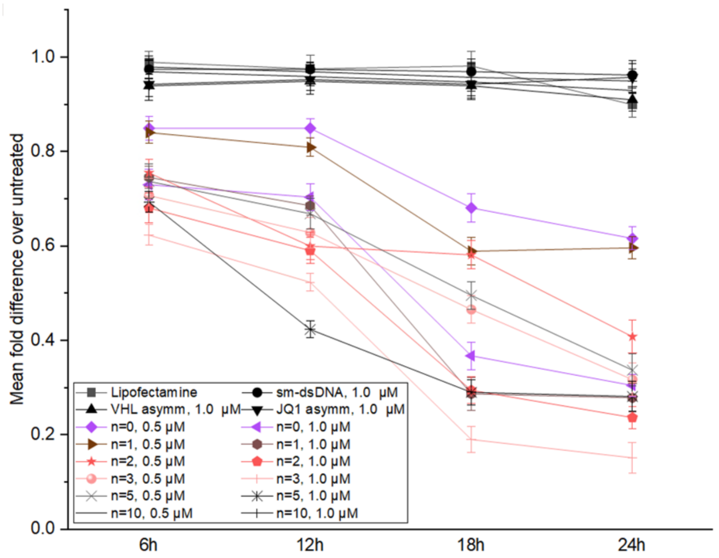

Supplementary Figure 22. Viability of the HEK293T upon treatment with OligoPROTAC of varying linker lengths and partial PROTAC control constructs evaluated by Cell-Titer Glo.

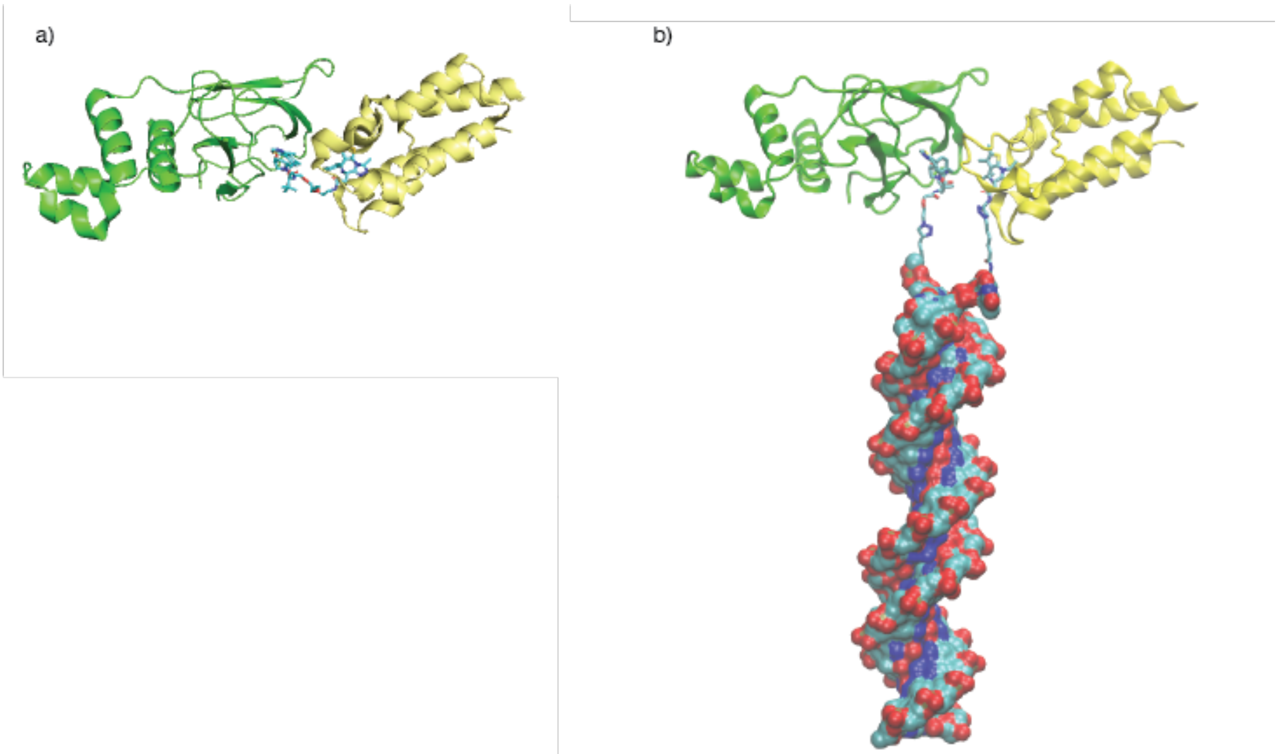

Supplementary Figure 23. Molecular model of OligoPROTAC, [n=3] compared to small molecular PROTAC, MZ1. a) Crystal structure, 5T35[3], modified to show MZ1, PROTAC warheads, VH032 and (+)-JQ1 in complex with BRD4^{BD2} (yellow) and pVHL (green). b) Molecular model of OligoPROTAC, [n=3] with VH032 and (+)-JQ1 warheads in complex with BRD4^{BD2} (yellow) and pVHL (green). The 23-bp duplex DNA stem is illustrated as a space fill model.

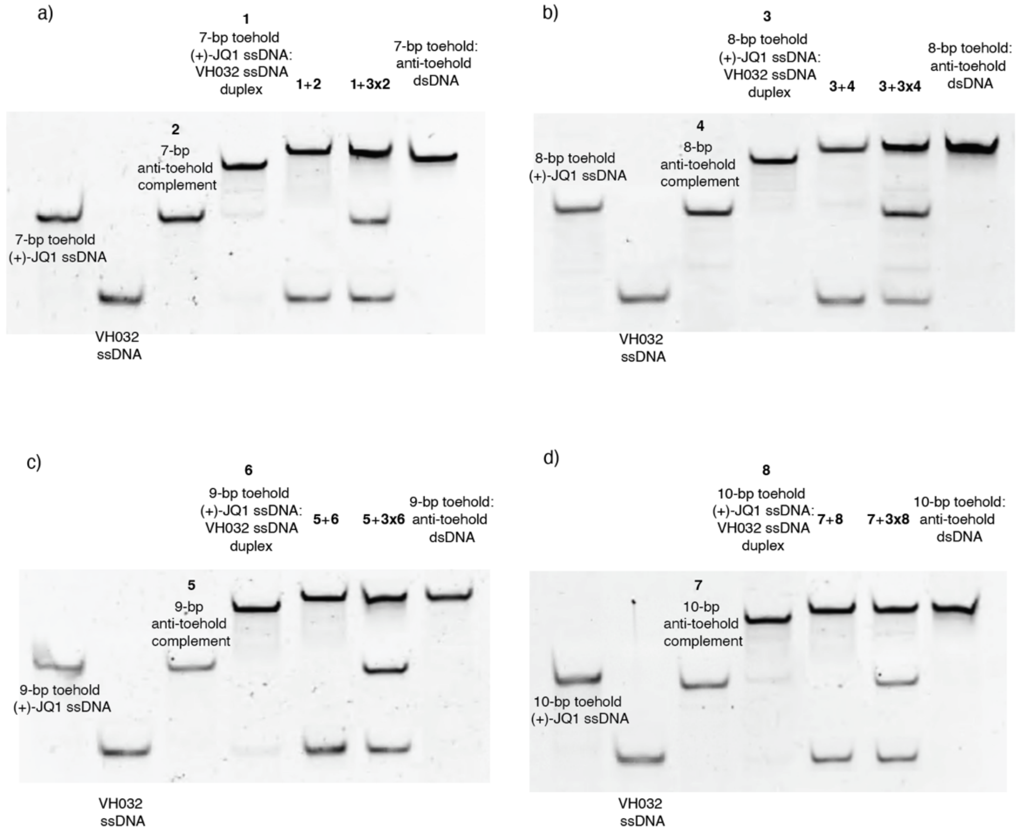

Supplementary Figure 24: Native PAGE gel for screening toehold sequences (7, 9, 10-base pair toehold, sequences in Supplementary Table 1) for toehold mediated strand displacement "off" switch for OligoPROTAC construct

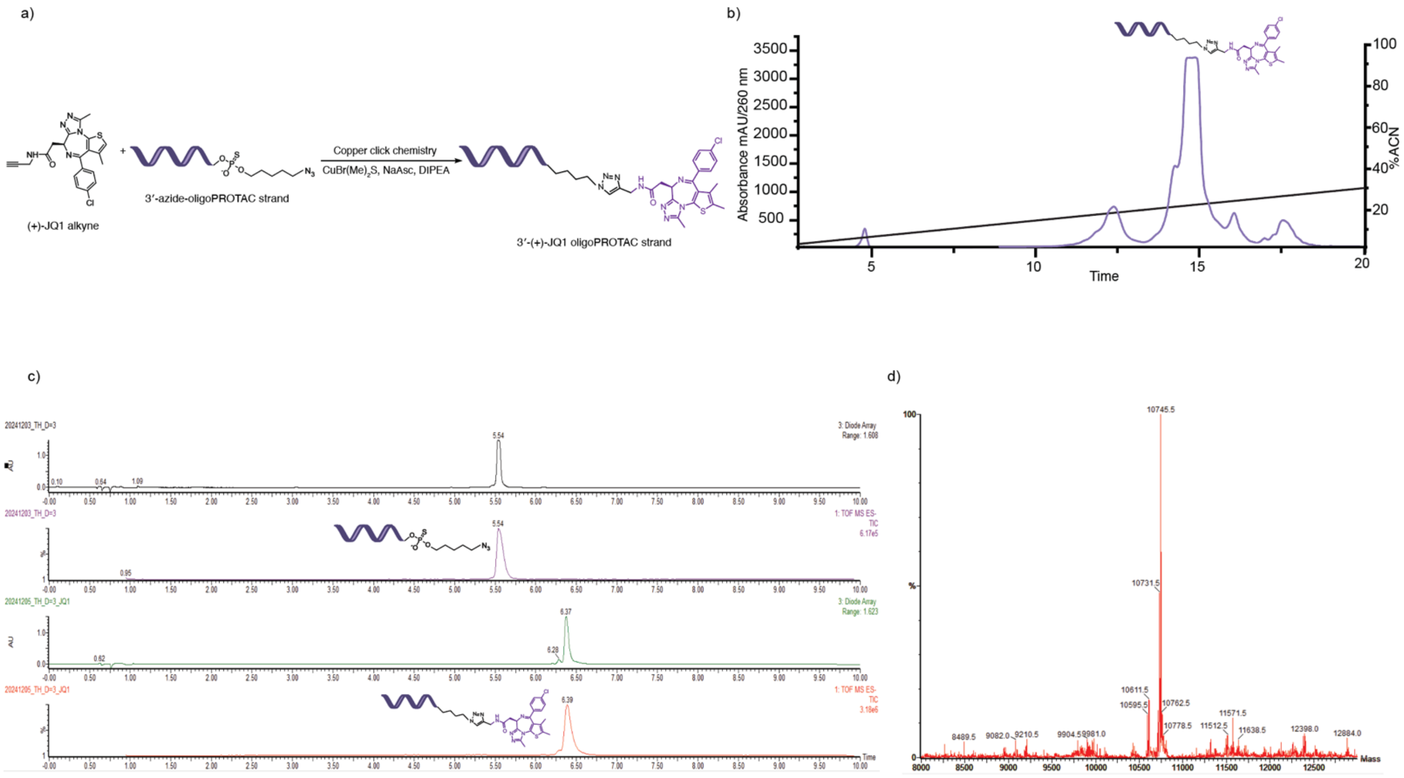

Supplementary Figure 25. Reaction and characterisation for toehold-(+)-JQ1 ssDNA OligoPROTAC, [n=3]. **a**) Reaction scheme for copper click conjugation of (+)-JQ1-alkyne with azide-toehold-OligoPROTAC, [n=3]. **b**) HPLC purification for toehold-(+)-JQ1 ssDNA OligoPROTAC, [n=3]. **c**) LC-MS characterisation for toehold-(+)-JQ1 ssDNA OligoPROTAC, [n=3]. **d**) Mass spectrum for HPLC-purified toehold-(+)-JQ1 ssDNA OligoPROTAC, [n=3].

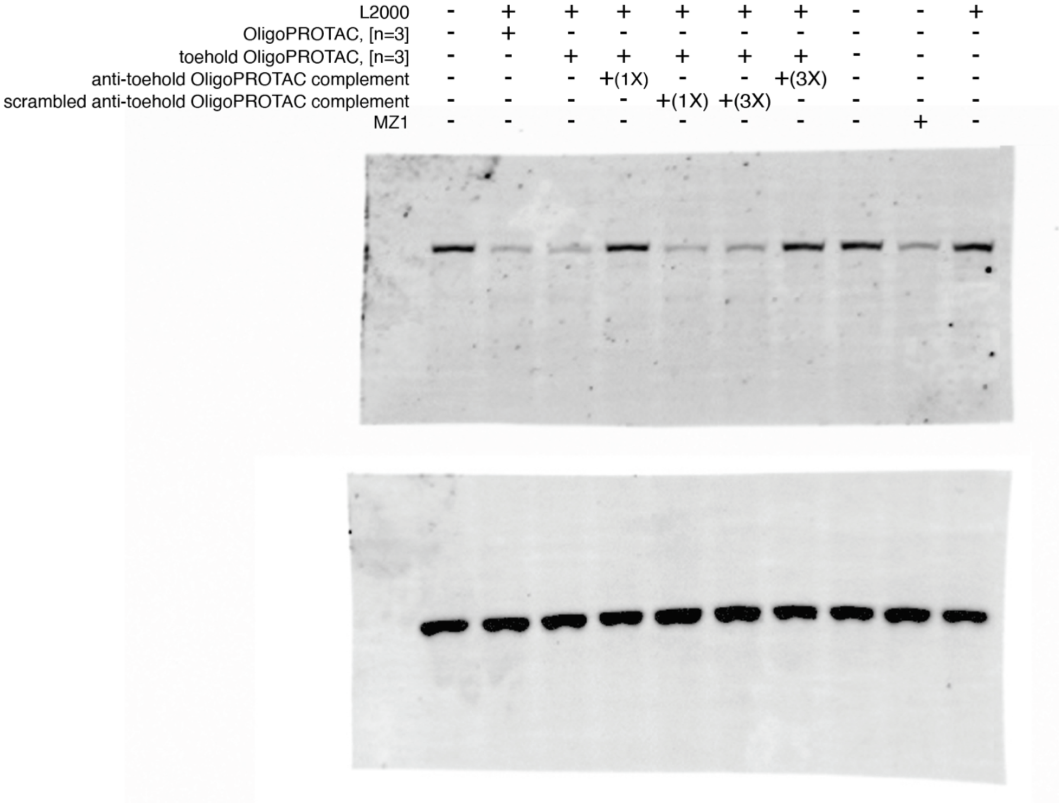

Supplementary Figure 26. Uncropped western blot of BRD4 levels upon treatment with toehold OligoPROTAC [n=3], along with anti-toehold complement and scrambled anti-toehold complement upon lipofectamine 2000 transfection in HEK293T cells.

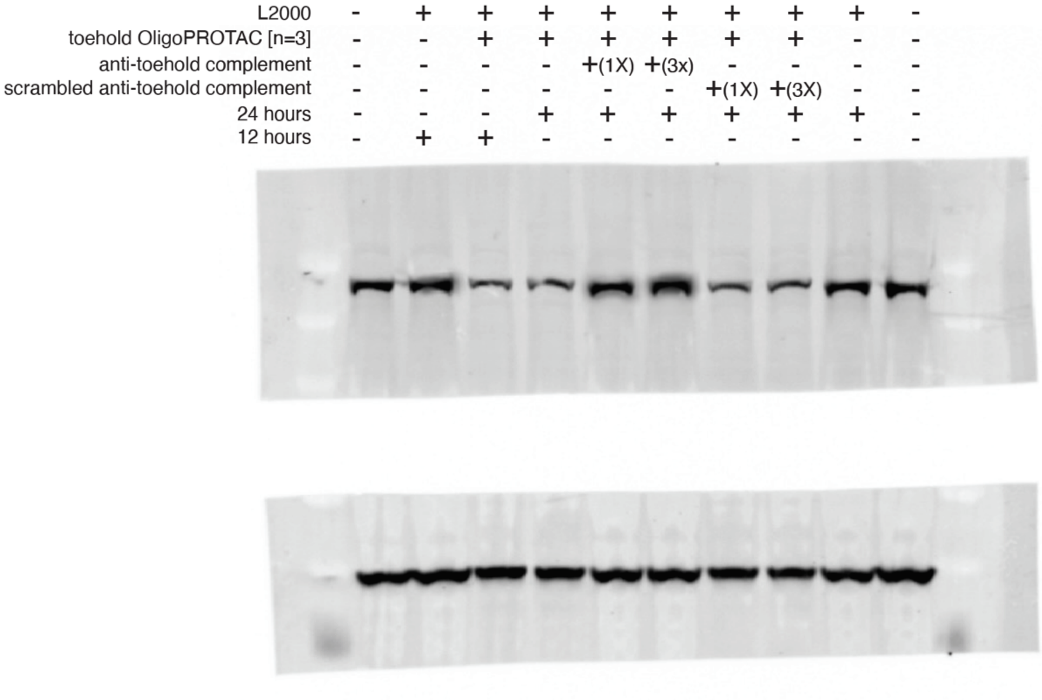

Supplementary Figure 27. Uncropped western blot of BRD4 levels upon treatment with toehold OligoPROTAC [n=3] for 12 hours, followed by anti-toehold complement and scrambled anti-toehold complement incubation for another 12 hours – harvest at 24 hours

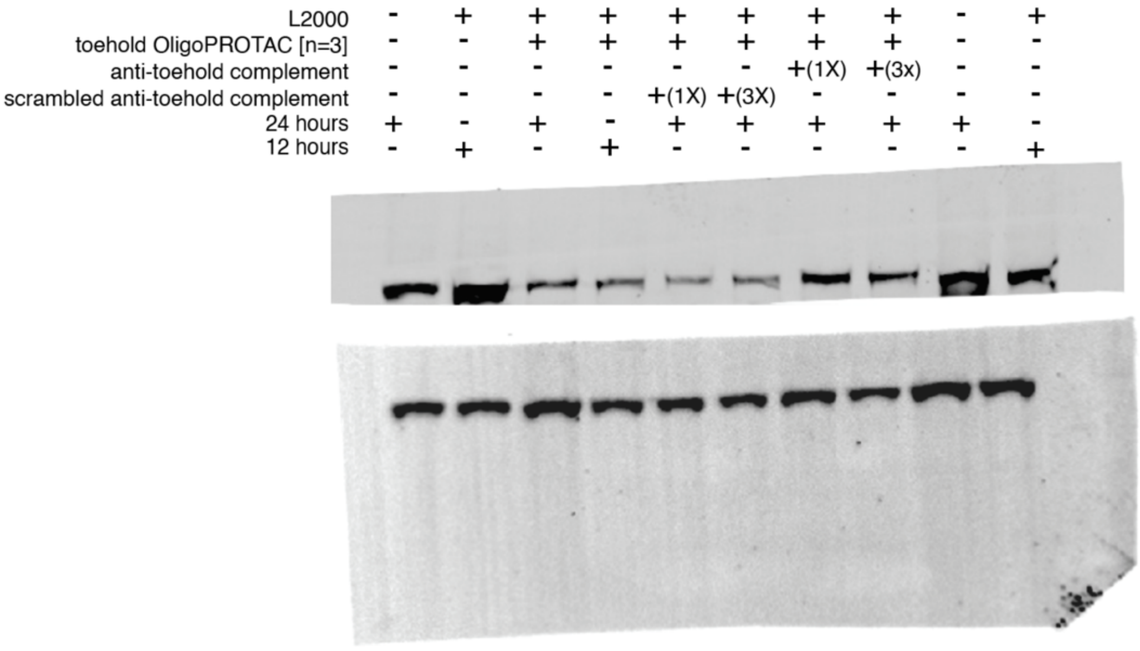

Supplementary Figure 28. Uncropped western blot of BRD4 levels upon treatment with toehold OligoPROTAC [n=3] for 12 hours, followed by anti-toehold complement and scrambled anti-toehold complement incubation for another 12 hours – harvest at 24 hours (biological replicate).

References

(1) He, S.; Dong, G.; Li, Y.; Wu, S.; Wang, W.; Sheng, C. Potent Dual BET/HDAC Inhibitors for Efficient Treatment of Pancreatic Cancer. *Angew Chem Int Ed Engl* **2020**, *59* (8), 3028-3032. DOI: 10.1002/anie.201915896 From NLM.
(2) Yoo, H.-D.; Shin, Y.-J.; Kim, S. J.; Kim, B. K.; Lee, E. M.; Shin, S. H.; Kim, Y. H.; Choi, S. W.; Bae, M. S.; Yang, D.; et al. Piperidinedione Derivative. **2023**, 1-45. From EPO.
(3) Gadd, M. S.; Testa, A.; Lucas, X.; Chan, K.-H.; Chen, W.; Lamont, D. J.; Zengerle, M.; Ciulli, A. Structural basis of PROTAC cooperative recognition for selective protein degradation. *Nature Chemical Biology* **2017**, *13* (5), 514-521. DOI: 10.1038/nchembio.2329.
(3) Wilkinson, A. C.; Ballabio, E.; Geng, H.; North, P.; Tapia, M.; Kerry, J.; Biswas, D.; Roeder, R. G.; Allis, C. D.; Melnick, A.; et al. RUNX1 is a key target in t(4;11) leukemias that contributes to gene activation through an AF4-MLL complex interaction. *Cell Rep* 2013, *3* (1), 116-127. DOI: 10.1016/j.celrep.2012.12.016. From NLM.
